# TranExamic Atomized for Pediatric post-Operative Tonsillectomy hemorrhage (TEAPOT): Study protocol for a pilot randomized controlled trial

**DOI:** 10.1371/journal.pone.0353841

**Published:** 2026-07-28

**Authors:** Andrew D. Meyer, Whitney Schwarz, Dylan Erwin, Marisa Earley, Venkata K. Yellepeddi, Thomas H. Chun, T. Charles Casper, Daniel K. Nishijima

**Affiliations:** 1 Department of Pediatrics, Long School of Medicine at the University of Texas Health Science Center, San Antonio, Texas, United States of America; 2 Department of Emergency Medicine, Long School of Medicine at the University of Texas Health Science Center, San Antonio, Texas, United States of America; 3 Department of Otolaryngology, Long School of Medicine at the University of Texas Health Science Center, San Antonio, Texas, United States of America; 4 Spencer Fox Eccles School of Medicine, University of Utah, Salt Lake City, Utah, United States of America; 5 Department of Emergency Medicine, Rhode Island Hospital/Hasbro Children’s Hospital and Brown University, Providence, Rhode Island, United States of America; 6 Department of Emergency Medicine, UC Davis School of Medicine, Sacramento, California, United States of America; PLOS: Public Library of Science, UNITED STATES OF AMERICA

## Abstract

Tonsillectomy is the second most common performed surgical procedure in children in the United States. Unfortunately, up to five percent of children return to emergency departments (ED) for post-tonsillectomy hemorrhage (PTH). To control PTH, most pediatric otolaryngologists return to the operating room (OR) for cauterization of the bleeding source. Retrospective studies suggest that nebulized tranexamic acid (TXA), an antifibrinolytic agent, can reduce the severity of post-tonsillectomy hemorrhage (PTH), however, its efficacy and safety have not yet been tested in randomized controlled trials. We designed the TranExamic Atomized for Pediatric post-Operative Tonsillectomy hemorrhage (TEAPOT) trial protocol to evaluate the feasibility of conducting a confirmatory clinical trial to evaluate the effects of TXA in children with PTH. We will randomize children presenting to the ED with secondary PTH to receive three 5-mL nebulized doses of either TXA (TXA 100 mg/ml) or placebo (normal saline). The following outcomes will be measured: feasibility (number of enrolled patients per site per year); protocol adherence (receipt of at least two nebulization doses); return to the operating room; pain reported on the FACES scale; parent and child anxiety assessed by the Patient-Reported Outcomes Measurement Information System (PROMIS) Anxiety measure; systemic and local (pharmacokinetic model derived) concentrations of nebulized TXA; coagulation biomarkers; and adverse events, including bleeding, thrombosis, and seizures. Our multicenter trial will provide important preliminary data on the feasibility and pharmacokinetics of nebulized TXA in preparation for a definitive clinical trial of children with secondary post-tonsillectomy hemorrhage. Registered at ClinicalTrials.gov ID# (NCT07565753) on May 4^th^, 2026, https://clinicaltrials.gov/study/NCT07565753?term=teapot&viewType=Card&rank=1

## Introduction

Over half a million tonsillectomies are performed in the United States every year. The national incidence of secondary post-tonsillectomy hemorrhage (PTH) is estimated to be as high as 3–5% and presentation varies from mildly darkened oral secretions to active hemoptysis [1–3]. The incidence maybe under-reported, because: 1) most of the studies are retrospective with varying criteria for defining PTH and 2) emergency department (ED) studies report high rates of re-bleeding in patients that previously presented with a normal examination or stable clot [4]. Previously reported non-operative interventions have failed to prevent the need to return to the operating room (OR) [5,6]. Among surveyed otolaryngologists (ENTs), 65–75% recommended that the standard treatment for post-tonsillectomy hemorrhage (PTH) is returning to the operating room (OR) for surgical control of bleeding [7]. Erwin *et al* published results of a retrospective cohort study documenting three doses of nebulized tranexamic acid (TXA), an antifibrinolytic, appears to be safe and beneficial for PTH; the study demonstrated a 44% reduction in the need for surgical re-establishment of hemostasis [8]. Moreover, this report documented reduced pharyngeal examination bleeding in response to nebulized TXA. Nebulized TXA is the first non-operative PTH intervention that has significantly decreased the need for anesthesia or an invasive procedure for upper airway bleeding in children [9,10]. While earlier reports found *intravenous* TXA to increase the seizure risk in pediatric patients [11], adverse neurological reactions caused by topical or local TXA are rare [12,13] and require further study.

Nebulization of TXA is an uncommon route of administration for antifibrinolytics, which are drugs that inhibit fibrinolysis, the breakdown of blood clots. Measuring the amount of local and systemic absorption of aerosolized drugs is challenging and subsequently understudied. Inhaled or intranasal medication can be rapidly absorbed across the large surface area of the respiratory tract epithelium. Some drugs absorbed into the pulmonary circulation enter directly into the systemic circulation via the pulmonary vein, possibly bypassing first-pass metabolism that occurs with oral drugs. Most investigations into the site of action for inhaled medications have relied on animal studies, since direct biopsy of the airway in humans poses significant risk [14]. Nonetheless, selecting an appropriate dose is critical to characterize drug absorption at both the local and systemic levels. Obtaining typical PK data in children is challenging due to the need for repeated blood sampling; however, physiologically-based PK (PBPK) modeling provides an alternative approach to estimate local and systemic concentrations [15]. These models have been shown to accurately predict the local and systemic PK of drugs across the respiratory epithelium after inhalation administration [16]. Accordingly, a pulmonary PBPK model can also be developed for TXA using physiological parameters of the respiratory system (pharynx, trachea, bronchi, bronchioles, etc.) together with drug-specific physicochemical and absorption, distribution, metabolism, and elimination (ADME) properties obtained from the literature. [17]. By leveraging this body of PK data on TXA, we aim to construct a PBPK model to predict both local and systemic absorption. The main advantage of this method to understand how an inhaled drug is absorbed and metabolized in the respiratory epithelium is use of only a few systemic samples. This technique has been used in multiple pediatric studies to predict the dosing response in various populations using different routes of administration [18,19].

Although nebulized TXA is effective in achieving hemostasis in adult and pediatric aerodigestive hemorrhage, there are only retrospective reports for its use in secondary PTH [8–10,20]. The ease of use and perception of success of nebulized TXA is growing in pediatric emergency departments [21]; a randomized clinical trial of this intervention compared to placebo is therefore necessary. We will conduct a randomized, blinded, multi-center pilot trial to determine the feasibility, protocol adherence, and drug absorption of nebulized TXA in children with post-tonsillectomy hemorrhage. This objective of this pilot study is to assess the feasibility of enrollment rates and the effectiveness of follow-up methods to inform the design and implementation of a larger trial.

## Materials and methods

### Study design

**Fig 1:**
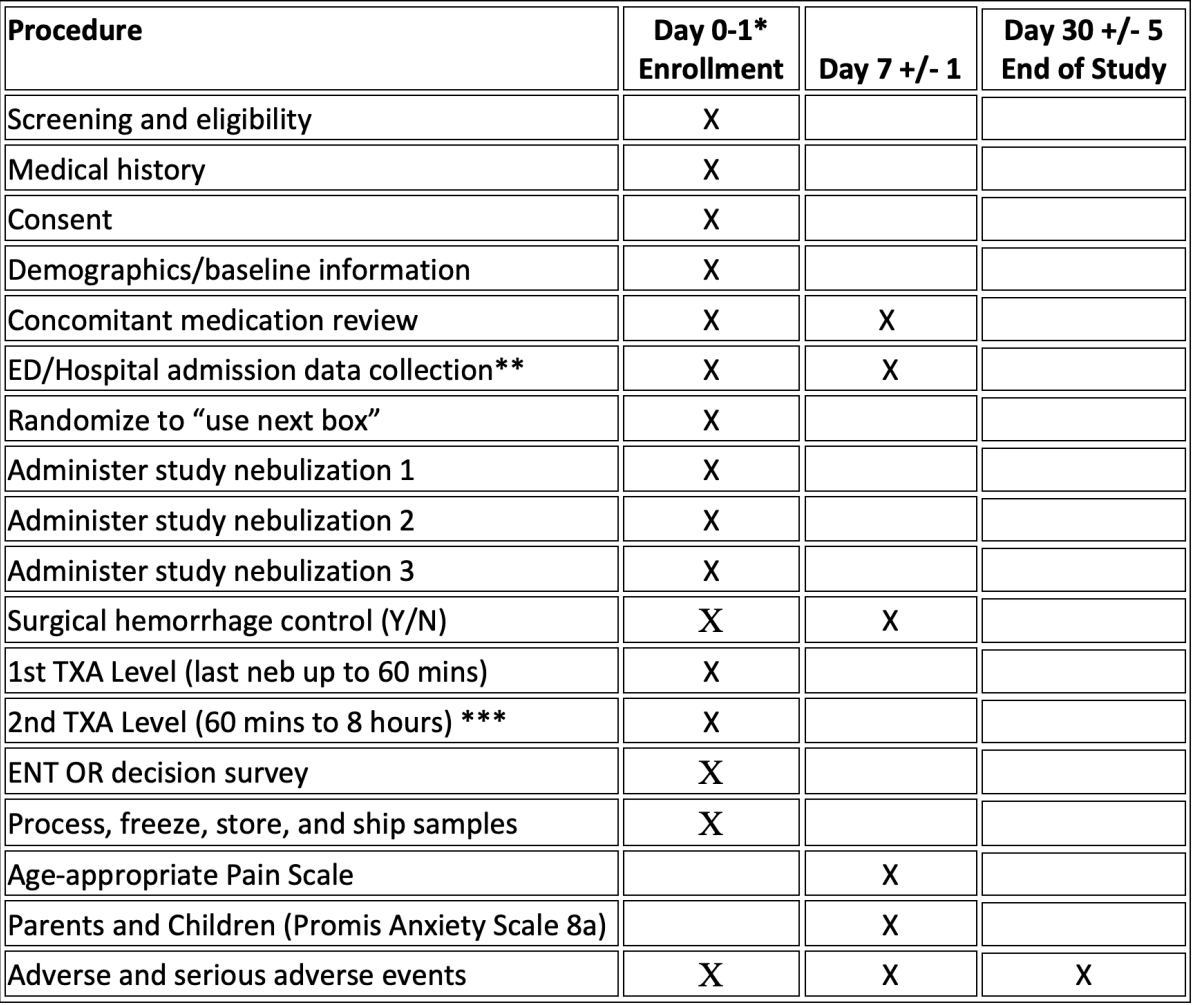
SPIRIT Schedule of Activities. *Randomization to 24 hours. **Should include the following, if collected per standard of care: vital signs (including height and weight), physical exam, ENT upper airway exam, lab data collection (coagulation labs, other routine lab tests), OR use, ENT exam & findings as relating to OR use (e.g., photo of tonsillar bed), blood product usage, final hospital disposition. At seven days, further, evaluate if participants had been readmitted for bleeding after an intervention. ***Time points must be separated by 60 to 90 minutes.

**Fig 2:**
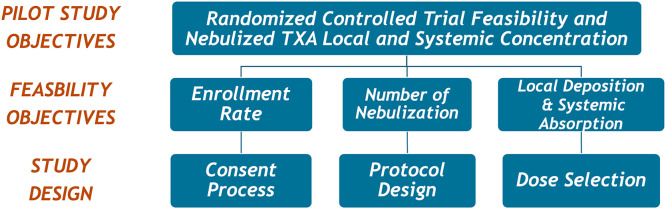
Study objectives of the TEAPOT Pilot Trial. TXA-Tranexamic acid.

The manuscript has been prepared in accordance with SPIRIT guidelines. A completed SPIRIT Schedule of Enrollment, Interventions, and Assessments is provided as Fig 1 [23]. To prepare for a confirmatory clinical trial, we designed a pilot, multi-center, randomized controlled, blinded pilot trial to assess the feasibility, safety, and the local and systemic absorption of nebulized TXA in children with PTH (Fig 2). Some ED-based trials have shown that pre-consenting (advance consent) may not be feasible for certain types of interventions and have not reliably improved enrollment rates [22]. Children 2–17 years old presenting to the ED with PTH will be randomized in a 1:1 ratio to three doses of nebulized TXA or placebo (saline).

### Study setting

The pilot study will be conducted at three pediatric academic medical centers in the United States: University Hospital/University of Texas Health Science Center at San Antonio (San Antonio, Texas), UC Davis Children’s Hospital (Sacramento, California), and Hasbro Children’s Hospital/Brown University Health (Providence, Rhode Island). Two of these sites, UC Davis Children’s Hospital and Hasbro Children’s Hospital, are members of the Pediatric Emergency Care Applied Research Network (PECARN). PECARN is the largest federally funded pediatric emergency medicine research network in the United States and consists of 18 pediatric emergency departments. PECARN reviewed and approved the protocol design and confirmed its willingness to support a future Phase III trial if the pilot study demonstrates feasibility. The participating sites were selected based on their established pediatric emergency medicine and otolaryngology programs, experience conducting multicenter clinical trials, and annual volumes of pediatric post-tonsillectomy hemorrhage presentations.

The initial three sites have dedicated pediatric EDs and an average PTH incidence of 3.8% and are committed to a blinded randomized trial with placebo to determine the true efficacy of nebulized TXA. Each site will work with its Patient Family Advisory Council and other community engagement groups to inform study design, family-centered outcomes, and feasibility. We will also engage ENT community partners and advocacy groups across the country to ensure diverse representation of individuals with PTH. Patients and the public were involved in the review of this research protocol. Specifically, the study design, objectives, and procedures were presented to the University Women and Children Hospital Pediatric Patient and Family Advisory Committee. The committee reviewed the protocol, including the consent process and the patient-centered outcomes, and expressed support for the study as presented without recommending any modifications. Following the completion of this pilot study, we plan to continue engaging with patient and family advisory councils to help shape the design and implementation of the subsequent larger Phase 3 trial.

### Inclusion and exclusion criteria

Children greater than or equal to 2 and less than 18 years of age with evidence of secondary PTH will be eligible for the study. Secondary PTH refers to bleeding at the surgical site that starts more than 24 hours after tonsillectomy. The number of missed eligibles, screen failures, and voluntary withdrawals will be tracked. Eligible patients may be missing because of research assistant unavailability, miscommunication, misapplication of screening criteria, or other factors. A waiver of consent will be obtained at each site (up to 100 patients) to review ED records to identify missed eligibles. Screen failures are defined as participants that consent to take part in the clinical trial but are later found to be ineligible. A minimal set of screen failure and missed eligibles information must ensure transparent reporting of screen failure participants to meet the Consolidated Standards of Reporting Trials (CONSORT) publishing requirements and to respond to queries from regulatory authorities. Minimal information includes demography, screen failure details, eligibility criteria, and any serious adverse event (SAE). See Tables 1 and 2 for specific inclusion and exclusion criteria.

**Table 1:** Inclusion Criteria for TEAPOT trial.

1. Received a tonsillectomy
2. Presents to the ED with secondary* post-tonsillectomy hemorrhage
3. Children between age of 2–17 years of age (i.e., before their 18^th^ birthday)

*Secondary post-tonsillectomy hemorrhage is defined as greater than 24 hours from their primary tonsillectomy operation (arrival in recovery/PACU).

**Table 2:** Exclusion Criteria for TEAPOT Trial.

1. Known and documented bleeding or clotting disorder
2. Known pregnancy
3. Patients who are wards of the state, as defined under 45 CFR 46.409(b)
4. Patients with known hypersensitivity or allergic response to tranexamic acid
5. Parents or guardians that cannot communicate in English or Spanish.
6. Intubation prior to enrollment
7. Previously enrolled patients

### Participant screening, consent, and assent

The study was approved by the University of Utah Institutional Review Board (IRB_00191036), which serves as the central IRB for all participating sites. Initial approval was granted on October 29, 2025, with a protocol amendment approved on April 9, 2026. Written informed consent will be obtained from the parent or legal guardian before enrollment, with assent obtained from children when appropriate. Before the start of the study and during its course, clinical research coordinators and ED clinicians will be educated about the trial and trained to identify eligible patients. We will screen patients and patients believed to be eligible will be discussed with the treating physicians. We will approach the patient’s legally authorized representative to obtain written informed consent. We will obtain assent from children who are able to understand the trial, which the investigators designate as greater than or equal to 7 years of age. The child will be given an age-appropriate explanation of the procedures to be used, their meaning to the child in terms of discomfort and inconvenience, and the general purpose of the research. The informed consent document will be provided to the parent or legal guardian, in addition to an age-appropriate information sheet for easier comprehension.

### Interventions

The intervention will not interfere with or supersede standard ED or ENT care for PTH. After determination of eligibility and obtaining informed consent, patients will be randomized to receive nebulized TXA or saline. The only change to ED PTH management is the addition of the nebulized study drug. All standard supportive care for post-tonsillectomy hemorrhage, including analgesics and antiemetics, is permitted. However, the administration of any other non-protocol hemostatic agents (e.g., aminocaproic acid, topical thrombin) is prohibited during the initial 24-hour study period unless deemed medically necessary by the clinical team for rescue purposes after the study intervention has been completed. The investigational product, TXA 100 mg/mL, is approved in the pediatric population for the treatment of hemorrhage post-tooth extraction, and for short-term treatment of hemophilia.[24] Participants will receive three nebulized doses of either TXA 500 mg (5 mL of TXA 100 mg/ml) or 5 mL of placebo (normal saline) using a PARI LC D Disposable Nebulizer or equivalent over 10–15 minutes each using 8 or greater liter/minute of gas flow. Our preliminary studies and experience suggest that nebulized TXA can clog standard nebulizers; optimal delivery is achieved when using a jet nebulizer such as the PARI LC D. The PARI LC D has fill volume of 6 mL. To deliver a total of 15 mL saline or 1500 mg TXA will require three consecutive nebulization.

The study drug will be discontinued if any of the following occur: suspected anaphylactic reaction, sudden precipitous respiratory failure, ENT taking the patient to the operating room, withdrawal of consent by the patient’s legal guardian or legally authorized representative, or discovery of new information which makes the patient ineligible to continue participation in the study. Both study patients and study team members are blinded to the interventional arm. Blinding is provided using identical study drugs, packaging and vial volumes. Given no reversal agent, treatment assignment will not be unblinded. Clinical teams will be asked to assume the patient received TXA.

### Randomization

The narrow time window for study intervention necessitates that randomization does not delay treatment. As such, the study intervention will be preassigned using a central randomization process. Prior to enrollment at each site, a study drug box having two vials (1000 mg per vial) of blinded study drug, nebulizer, and mask with a numeric identification code corresponding to the treatment assignment will be designated as the “Use Next Box.” Eligible patients will be randomized into one of the two arms in a 1:1 ratio (TXA vs saline). Permuted-block randomization will be used to maintain balanced allocation of participants to the TXA treatment group. A patient is considered enrolled when randomization occurs.

### Pharmacokinetic and pharmacodynamic testing

**Fig 3:**
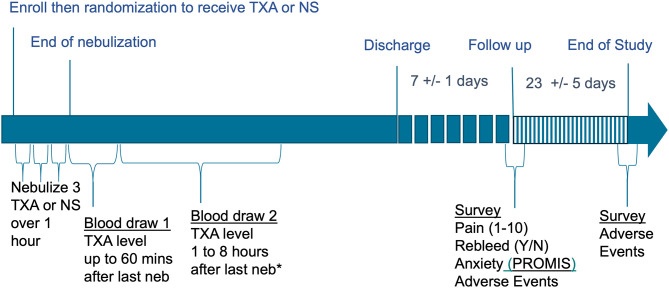
Study Timeline (*at least six minutes after last neb). PROMIS = Patient-Reported Outcomes Measurement Information System Anxiety Short Form. PROMIS Anxiety surveys will be administered to parents and age-appropriate child participants at the designated study time points. Adverse events will be collected through a phone call at 30 days ± 5 days. TXA-Tranexamic acid, NS-normal saline, neb-nebulization.

PK samples will be collected within sixty minutes after completion of the last nebulized treatment received. A second point collection will occur between sixty minutes and eight hours after last nebulization completion, separated from the previous time point by at least sixty minutes (Fig 3). Results from TXA level measurements will be used for research purposes only and not available to clinicians. Serum TXA levels will be used to develop a TXA PK model and determine population variability. A base model will be developed to determine the best-fit compartmental model, distribution, and elimination kinetics. Stochastic models will be used to evaluate between-subject variability in PK parameters. Serum TXA concentrations will use 200 microliters of plasma measured by an ultra-high performance liquid chromatography tandem mass spectrometry method specifically developed for sensitive and precise analysis of low TXA concentrations at the U.S. Army Institute for Surgical Research. The remaining 200 microliters will be used to measure pharmacodynamics effects of nebulized TXA in the plasma. Less than 300 μL of plasma is sufficient to run all ELISA kits measuring plasmin-antiplasmin (PAP) complexes, d-dimer, thrombin-antithrombin (TAT) complexes, prothrombin fragment F1 + 2 (F1 + 2), and plasminogen activator inhibitor-1 (PAI-1). Parameters are performed at a central laboratory to eliminate inter-assay variability and to standardize testing.

### Outcomes

We will collect vital signs (including height/weight), physical exam, upper airway and pulmonary examination, and findings as relating to OR use (*e.g.,* photo of tonsillar bed), total blood products (mL/kg) transfused over the initial 24 hours of care, blood product usage, final hospital disposition. Patient-reported outcome measures (PROMs) will be collected at 7 (+/- 1) days after randomization. This will include pain, assessed using an age-appropriate scale (e.g., Wong-Baker FACES Pain Rating Scale [25]), and anxiety. Anxiety will be measured using the Patient-Reported Outcomes Measurement Information System (PROMIS) Anxiety short forms [26]. For parents and for children aged 8 years and older, the PROMIS Anxiety 8a short form will be used. For children under 8, parents will complete the PROMIS Anxiety Parent Proxy short form on their child’s behalf. Anxiety outcomes will be reported as standardized T-scores. Safety outcomes will be assessed on day 1, 7, and 30 after randomization via review of the electronic medical record and participant outreach via telephone or email. Safety outcomes include, but are not limited to, thromboembolic events, seizures, and rebleeding of tonsillectomy bed requiring emergency or clinic visit.

### Data management and quality plan

The study data coordinating center (the Emergency Medical Services for Children Data Center at the University of Utah) will create the electronic data capture system and worksheets. We will maintain study worksheets and documents in locked file cabinets in locked offices or using encrypted servers at each site. A trained site monitor will conduct web-based site-monitoring to provide a written report of their visits during the study period that ensures regulatory compliance and patient safety, and to monitor the quality of data collected at each enrolling site. A Data and Safety Monitoring Board (DSMB), approved by the funding agency, will advise the sponsors and principal investigators regarding serious adverse events and other patient safety issues. The DSMB will include an ethicist, a pediatric ENT, a pediatric hematologist, a patient advocate, and a pediatric emergency medicine physician.

### Study status and timeline

At the time of manuscript submission, participant recruitment had not yet begun, and no study outcome data had been collected. Central multi-site IRB approval was obtained through the University of Utah Institutional Review Board, and site activation activities, personnel training, investigational pharmacy preparation, and database development have been completed at University Hospital/University of Texas Health Science Center at San Antonio, UC Davis Children’s Hospital, and Hasbro Children’s Hospital.

Participant recruitment is anticipated to begin in July 2026 and continue through June 2027. Follow-up and data collection are expected to be completed by July 2027. Primary feasibility, pharmacokinetic, and safety analyses are anticipated during the second half of 2027, with dissemination of study results thereafter.

### Statistical considerations

The primary objective of this trial is to assess the feasibility of study procedures; it is not powered to detect differences in clinical outcomes. Feasibility outcomes include the ratio of enrolled to screened participants and the proportion of participants receiving at least two nebulization doses. We set an enrollment goal of 0.6 eligible patients per site per month, which would support the feasibility of a subsequent confirmatory trial evaluating the safety and efficacy of nebulized TXA for the primary outcome (need for surgical intervention: yes/no).

Sample size estimates for a future Phase III trial are based on a conservative effect size of 0.20, informed by a large retrospective study showing an absolute risk reduction of 24% [10] and a 2024 meta-analysis reporting a risk ratio of 0.55 [27]. Using a one-tailed Fisher’s exact test stratified by site, with 90% power and a one-sided α of 0.025, this corresponds to approximately 130 participants per arm (260 total). To account for stratification and potential P data, we increased the target by 15% to 150 per arm (300 total). Conducting this trial across 11 sites over 4 years would therefore require an average recruitment rate of 0.6 participants per site per month. For the primary feasibility outcomes, all analyses will be based on available data, and the proportions of missing data for each outcome will be reported. For secondary clinical outcomes, analyses will follow the intention-to-treat (ITT) principle. The extent and pattern of missing data will be summarized for all endpoints. If the rate of missing data is less than 5% for any given outcome, a complete case analysis will be performed. Should missingness exceed this threshold, we will assess the likely mechanism of missingness and employ appropriate methods, such as multiple imputation, under the assumption that data are missing at random (MAR).

We will initially evaluate the feasibility of enrolling patients with parent or legal guardian written informed consent only. If these consent procedures are not feasible (*i.e.,* time to consent in actively hemorrhaging child is superseded by providers’ decision to get treatment started, or patient is hastily taken to the OR), we will apply to use exception from informed consent (EFIC) procedures. We will collect detailed information on missed eligible patients to evaluate the feasibility of consent processes to determine when to initiate EFIC procedures.

We will select the PBPK model that best characterizes the data using individual and population acceptance criteria. To apply the acceptance criteria, we will simulate plasma drug concentrations for everyone using the PBPK model and will compare predictions with observed concentrations. We will assess model bias by calculating the average fold error between predicted and observed concentrations: AFE = 10[1/N∑log(predicted/observed)]. The AFE indicates model under- (AFE < 1) or over-prediction (AFE > 1) when compared with observed values of the individual. We will assess model precision by calculating the absolute average fold error: AAFE = 10[1/N∑|log(predicted/observed)] for each curve. We will consider <30% bias (AFE 0.7–1.3) and ≤2-AAFE reasonable predictions. We will assess population predictability by generating a prediction interval (5^th^ to 95^th^ percentile) of drug concentrations per time point for the population and quantifying the number of observed concentrations that fall outside of the prediction interval. The model will be accepted if 90% of observed concentrations fall within the prediction interval.

### Supporting information

The complete study protocol is available as S1 File. The completed SPIRIT 2025 checklist is provided as S2 File, and the completed WHO Trial Registration Dataset checklist is provided as S3 File.

## Discussion

Non-operative management of airway and upper GI tract bleeding is of vital interest to pediatric surgeons, emergency, and intensive care providers. General anesthesia increases the risk of neurotoxicity in children, suggesting a need to avoid surgical management of PTH when safely possible [28]. The primary objective is to start with a well-designed pilot trial to assess the overall feasibility of conducting a subsequent, confirmatory clinical trial evaluating nebulized TXA to reduce the need for a surgical intervention for secondary PTH. We chose to begin with a rigorously designed pilot trial using all procedures planned for the definitive multicenter trial to evaluate feasibility before initiating a fully powered Phase III study. A smaller pilot trial can give go/no-go information on study design, outcome measurements, and feasibility to progress to a larger phase III trial while minimizing the risks to special populations such as children. Critical information that this pilot trial will provide include number of eligible patients at representative PECARN study sites, feasibility of timely consent and enrollment procedures, protocol adherence, and variability of care.

Systemic administration of TXA achieves hemostasis both intra- and post-operatively in orthopedic, dental, cardiovascular, and trauma literature [29]. However, systemic reviews report that intravenous TXA may not be effective when administered prophylactically or perioperatively for PTH prevention [30]. Additionally, large retrospective studies document that intravenous TXA can have significant delayed-onset adverse events including an increase in seizures, thromboembolic events, renal insufficiency, and changes in vision [11]. With equivocal outcomes and potential risks associated with systemic TXA administration, topical application of TXA may have fewer side effects and more efficacious due to increased airway and upper GI tract tissue exposure of inhaled drug formulations. A verified pulmonary PBPK model can determine the range of safe doses for a confirmatory phase III trial. A pulmonary PBPK model incorporates all known intravenous, oral, and local TXA administration PK and pulmonary physiology data. This model necessitates verification from only a small number of systemic samples compared to traditional pharmacokinetic studies which require multiple samples using different doses and ages of patient. A verified model can then be used to predict the drug dose with the lowest systemic absorption to minimize side effects for the phase II trial. There has been a significant increase in the use of physiologically based pharmacokinetic (PBPK) models during the past 20 years; it has been increasingly accepted by regulatory agencies to bring pediatric drugs to the market [31,32]. Pulmonary PBPK models have led to significant insights into the development of new inhaled formulations of drugs such as Fosfomycin as broad-spectrum inhaled drug for pneumonia [33].

Pediatric clinical trials are challenging compared to similar trials in adults given their unique obstacles. They may have a smaller patient pool requiring multi-center recruitment, variability in local ethics committee reviews of child consent processes, and the need for many different administrative tasks including Institutional Review Board approvals and consortium agreements. This and the inability to identify all the potential obstacles needed to optimize trial design have led to premature closure, or lack of completion, of several phase III pediatric clinical trials. In an analysis of 413 National Institutes of Health (NIH)-sponsored pediatric RCTs, Rees *et al* reported 64% were prospectively registered, 13% had submitted results to ClinicalTrials.gov within 12 months of completing the trial, 54% had published results by 48 months after grant completion, and 41% of the results were neither published nor posted on ClinicalTrials.gov [34]. In an earlier study of 559 trials registered between 2008–2010, difficulty with patient accrual (37%) was the most common cited reasons for discontinuation [35]. The development of networks such as the Children’s Oncology Group and PECARN have had increased success in publication of trials with their scrutiny of trial design and pilot testing to ensure adequate enrollment [36]. Furthermore, NIH and networks are beginning to apply gold standards to trial design, which we have done with TEAPOT including the application of checklists (e.g., SPIRIT), pre-registering the trial with ClinicalTrials.gov (NCT07565753), and working with a pediatric experienced and well-established data coordinating center [37]. **The completed WHO Trial Registration Dataset checklist is provided as** S3 File.

Limitations exist with the design of the pilot trail. Since publication of the first pediatric case study in 2018 [38], the use of off-label nebulized TXA by pediatric EM providers has increased significantly. This increase may point to a growing belief that nebulized TXA benefits outweigh its risk and it should not be studied in a randomized controlled trial. To understand this, we conducted a 2024 survey of 134 EM providers and 110 ENT providers, documenting that most institutions did not have clinical practice guidelines for the management of PTH (73.1% none, 10.4% unsure) [39]. Additionally, 64% of EM physicians have tried or will try nebulized TXA compared to 28.6% of ENT physicians (p < 0.001, publication pending). Moreover, over 80% of ENT respondents wanted more high-quality studies to evaluate non-operative interventions such as nebulized TXA and would prefer observation instead. Although PECARN has been successful in obtaining consent in emergency situations, the process may delay treatment and reduce enrollment and protocol adherence in the confirmatory trial. Missed patients who are eligible will therefore be tracked closely to determine whether we need to implement federal exception from informed consent (EFIC) procedures. Lastly, the results of the pilot trial may not be generalizable to a much larger number of clinical sites for the subsequent confirmatory study. To better anticipate potential barriers for subsequent trial, the pilot trial is being conducted at three clinical sites that bring their own demographic, geographic, and clinical diversity to the trial.

## Conclusions

In conclusion, the TEAPOT pilot trial will be the first randomized trial to assign nebulized TXA in children with secondary PTH. Results from this pilot will provide crucial preliminary information to conduct a confirmatory trial.

## Supporting information

S1 File Orginal Protocol. (DOCX)

S2 File Spirit Checklist. (DOCX)

S3 File WHO Trial Registration Checklist. (DOCX)
