# Supplementary material for: TranExamic Atomized for Pediatric post-Operative Tonsillectomy hemorrhage (TEAPOT): Study protocol for a pilot randomized controlled trial
Source: PLoS One. 2026 Jul 28;21(7):e0353841. doi: 10.1371/journal.pone.0353841 (PMC13411930; doi:10.1371/journal.pone.0353841)
Supplement: S1 File — (DOCX) [file pone.0353841.s001.docx]

**TranExamic Atomized for Pediatric post-Operative Tonsillectomy hemorrhage (TEAPOT):**

**A Pilot and Feasibility Study**

**Unique Protocol Identification Number: 145749-0006**

**National Clinical Trial (NCT) Identified Number: NCT06580509 & NCT07565753**

**Single Site Principal Investigator: Andrew D. Meyer MD, MS**

**Multiple Site Principal Investigators: Andrew D. Meyer, MD, MS**

**and Daniel Nishijima MD, MAS**

**IND Sponsor: Andrew D. Meyer MD, MS**

**Funded by:** This work was supported by the National Heart, Lung, and Blood Institute of the National Institutes of Health under award 1R34HL177446-01A1 to ADM, and by the 2024 University of Texas Health Science Center at San Antonio Clinical Pilot Award to ADM. The funders had no role in study design, data collection and analysis, decision to publish, or preparation of the manuscript.

**Version Number: 3.10 February 27, 2026**

Sponsoring Nodes: San Francisco-Oakland Providence and Atlanta Research Collaborative (SPARC) and Pediatric Research in Injuries and Medical Emergencies (PRIME)

Impact Statement: Bleeding after a tonsillectomy is one of the most common post-operative problems in children. Nebulized tranexamic acid (TXA) may be a safe and non-invasive therapy to decrease bleeding. If effective, nebulized TXA may improve child health by preventing the related immediate risks of a second operation, hospital readmission, and blood transfusions. Avoidance of a second surgery is beneficial as it avoids long-term risks of anesthesia, cauterization, anxiety, and pain.

Revisions:

1. April 2021 Changes after Utah DCC Discussions and Teleconferences
2. May 2021 Amendments by principal investigators
3. Oct 2021: Revisions from steering and subcommittees
4. Nov 2021: Revisions from HRSA
5. Dec 2021: TEAPOT Steering Committee endorsed protocol
6. Feb 2024: Preparation of grant submission and amendment to FDA
7. Jan 2025: FDA amendment of protocol for single site study with 21-CFR-11 waiver
8. Feb 2026: Amendment of protocol for multi-site study updated literature and simplified parent and child surveys (PROMIS).

**Table of Contents**

[Statement of Compliance 5](#_Toc188873310)

[1 Protocol Summary 6](#_Toc188873311)

[1.1 Synopsis 6](#_Toc188873312)

[1.2 Schema 7](#_Toc188873313)

[1.3 Schedule of Activities (SoA) 8](#_Toc188873314)

[2 Introduction 9](#_Toc188873315)

[2.1 Study Rationale 9](#_Toc188873316)

[2.2 Background 10](#_Toc188873317)

[2.3 Risk/Benefit Assessment 11](#_Toc188873318)

[2.3.1 Known Potential Risks 11](#_Toc188873319)

[2.3.2 Known Potential Benefits 12](#_Toc188873320)

[2.3.3 Assessment of Potential Risks and Benefits 13](#_Toc188873321)

[3 Objectives and Endpoints 14](#_Toc188873322)

[4 Study Design 16](#_Toc188873323)

[4.1 Overall Design 16](#_Toc188873324)

[4.2 Scientific Rationale for Study Design 16](#_Toc188873325)

[4.3 Justification for Dose 17](#_Toc188873326)

[4.4 End of Study Definition 18](#_Toc188873327)

[5 Study Population 19](#_Toc188873328)

[5.1 Inclusion Criteria 19](#_Toc188873329)

[5.2 Exclusion Criteria 19](#_Toc188873330)

[5.3 Lifestyle Considerations 19](#_Toc188873331)

[5.4 Screen Failures 20](#_Toc188873332)

[5.5 Strategies for Recruitment and Retention 20](#_Toc188873333)

[6 Study Intervention 23](#_Toc188873334)

[6.1 Study Intervention(s) Administration 23](#_Toc188873335)

[6.1.1 Study Intervention Description 23](#_Toc188873336)

[6.1.2 Dosing and Administration 23](#_Toc188873337)

[6.2 Preparation/Handling/Storage/Accountability 23](#_Toc188873338)

[6.2.1 Acquisition and accountability 23](#_Toc188873339)

[6.2.2 Formulation, Appearance, Packaging, and Labeling 24](#_Toc188873340)

[6.2.3 Product Storage and Stability 24](#_Toc188873341)

[6.2.4 Preparation 24](#_Toc188873342)

[6.3 Measures to Minimize Bias: Randomization and Blinding 25](#_Toc188873343)

[6.4 Study Intervention Compliance 25](#_Toc188873344)

[6.5 Concomitant Therapy 26](#_Toc188873345)

[6.5.1 Rescue Medicine 26](#_Toc188873346)

[7 Study Intervention Discontinuation and Participant Discontinuation/Withdrawal 27](#_Toc188873347)

[7.1 Discontinuation of Study Intervention 27](#_Toc188873348)

[7.2 Participant Discontinuation/Withdrawal from the Study 27](#_Toc188873349)

[7.3 Lost to Follow-Up 27](#_Toc188873350)

[8 Study Assessments and Procedures 28](#_Toc188873351)

[8.1 Efficacy Assessments 28](#_Toc188873352)

[8.2 Safety and Other Assessments 28](#_Toc188873353)

[8.2.1 Safety OUTCOMES 28](#_Toc188873354)

[8.2.2 HOSPITAL DATA COLLECTION and Patient-related OUTCOMES 28](#_Toc188873355)

[8.2.4 FOLLOW UP DATA COLLECTION 31](#_Toc188873356)

[8.3 Adverse Events and Serious Adverse Events 33](#_Toc188873357)

[8.3.1 Definition of Adverse Events (AE) 33](#_Toc188873358)

[8.3.2 Definition of Serious Adverse Events (SAE) 34](#_Toc188873359)

[8.3.3 Classification of an Adverse Event 34](#_Toc188873360)

[8.3.4 Time Period and Frequency for Event Assessment and Follow-Up 35](#_Toc188873361)

[8.3.5 Adverse Event Reporting 36](#_Toc188873362)

[8.3.6 Serious Adverse Event Reporting 36](#_Toc188873363)

[8.3.7 Reporting Events to Participants 37](#_Toc188873364)

[8.4 Unanticipated Problems 37](#_Toc188873365)

[8.4.1 Definition of Unanticipated Problems (UP) 37](#_Toc188873366)

[8.4.2 Unanticipated Problem Reporting 37](#_Toc188873367)

[8.4.3 Reporting Unanticipated Problems to Participants 38](#_Toc188873368)

[9 Statistical Considerations 39](#_Toc188873369)

[9.1 Statistical Hypotheses 39](#_Toc188873370)

[9.2 Sample Size Determination 39](#_Toc188873371)

[9.3 Populations for Analyses 39](#_Toc188873372)

[9.4 Statistical Analyses 40](#_Toc188873373)

[9.4.1 General Approach 40](#_Toc188873374)

[9.4.2 Analysis of the Primary Efficacy Endpoint(s) 40](#_Toc188873375)

[9.4.3 Analysis of the Secondary Endpoint(s) 40](#_Toc188873376)

[9.4.4 Safety Analyses 40](#_Toc188873377)

[9.4.5 Baseline Descriptive Statistics 40](#_Toc188873378)

[9.4.6 Planned Interim Analyses **Error! Bookmark not defined.**](#_Toc188873379)

[10 Supporting Documentation and Operational Considerations 42](#_Toc188873380)

[10.1 Regulatory, Ethical, and Study Oversight Considerations 42](#_Toc188873381)

[10.1.1 Informed Consent Process 42](#_Toc188873382)

[10.1.2 Study Discontinuation and Closure 43](#_Toc188873383)

[10.1.3 Confidentiality and Privacy 44](#_Toc188873384)

[10.1.4 Future Use of Stored Specimens and Data 44](#_Toc188873385)

[10.1.5 Key Roles and Study Governance 45](#_Toc188873386)

[10.1.6 Safety Oversight 45](#_Toc188873387)

[10.1.7 Clinical Monitoring 46](#_Toc188873388)

[10.1.8 Quality Assurance and Quality Control 48](#_Toc188873389)

[10.1.9 Data Handling and Record Keeping 50](#_Toc188873390)

[10.1.10 Protocol Deviations 53](#_Toc188873391)

[10.1.11 Publication and Data Sharing Policy 53](#_Toc188873392)

[10.1.12 Conflict of Interest Policy 53](#_Toc188873393)

[10.2 Additional Considerations 54](#_Toc188873394)

[10.2.1 FOOD AND DRUG ADMINISTRATION 54](#_Toc188873395)

[10.2.2 HEALTH INSURANCE PORTABILITY AND ACCOUNTABILITY ACT 54](#_Toc188873396)

[10.2.3 INCLUSION OF WOMEN AND MINORITIES 54](#_Toc188873397)

[10.2.4 CLINICALTRIALS.GOV REQUIREMENTS 54](#_Toc188873398)

[10.3 Abbreviations 54](#_Toc188873399)

[10.4 Protocol Amendment History 56](#_Toc188873400)

[11 References 57](#_Toc188873401)

[References 57](#_Toc188873402)

# Statement of Compliance

The trial will be conducted in accordance with International Council on Harmonization Good Clinical Practice (ICH GCP), applicable United States (US) Code of Federal Regulations (CFR), National Heart Lung and Blood Institute, and the University of Texas Health Science Center at San Antonio (UTHSCSA) and the following:

- - United States (US) Code of Federal Regulations (CFR) applicable to clinical studies (45 CFR Part 46, 21 CFR Part 50, 21 CFR Part 56, 21 CFR Part 312, and/or 21 CFR Part 812)

The Principal Investigators will assure that no deviation from, or changes to the protocol will take place without prior agreement from the funding agency and documented approval from the Institutional Review Board (IRB), and the Investigational New Drug (IND) or sponsor (UTHSCSA), if applicable, except where necessary to eliminate an immediate hazard(s) to the trial participants. All personnel involved in the conduct of this study have completed Human Subjects Protection and ICH GCP Training.

Investigators and clinical trial site staff who are responsible for the conduct, management, or oversight of clinical trials have completed Human Subjects Protection and ICH GCP Training. The protocol, informed consent form(s), recruitment materials, and all participant materials will be submitted to the IRB for review and approval. Approval of both the protocol and the consent form(s) must be obtained before any participant is consented. Any amendment to the protocol will require review and approval by the IRB before the changes are implemented to the study. All changes to the consent form(s) will be IRB approved; a determination will be made regarding whether a new consent needs to be obtained from participants who provided consent, using a previously approved consent form.

Investigator’s Agreement: I have read and understand the contents of this clinical protocol for Protocol UT Health San Antonio and will adhere to the study requirements as presented, including all statements regarding confidentially. In addition, I will conduct the study in accordance with current international conference on harmonization (ICH) guidance, Good Clinical Practice (GCP) guidance, the Declaration of Helsinki, US Food and Drug Administration (FDA) regulations and local IRB and legal requirements.

# 1 Protocol Summary

## 1.1 Synopsis

| **Title:** | TranExamic Atomized for Pediatric post-Operative Tonsillectomy hemorrhage: A Pilot and Feasiblity Study |  |  |  |
| --- | --- | --- | --- | --- |
| **Study Description:** | This study starts as single site pilot study, randomized, double-blinded controlled trial for 12 patients. Then dependent on funding, the study will be a multi-center, randomized, double-blinded, placebo-controlled trial. It is a parallel arms pilot trial randomizing up to 30 patients. |  |  |  |
| **Objectives:** | \| Primary Objective: \| The primary objectives of this pilot study are:   1. Enroll 0.6 patient's/site/month with PTH and 2. Evaluate the ability to nebulize at least two doses of TXA to children with PTH and 3. Determine the indirect local and systematic concentrations of nebulized TXA in children 4. Adverse events will be assessed 30 +/- 5 days from randomization \| \| \| \| --- \| --- \| --- \| --- \| \| Secondary Objectives: \| The secondary objectives of this pilot study are:   1. Determine the number and rationale for PTH patients to return to the OR; and 2. Determine the number of blood transfusions and estimated blood loss and 3. Determine the recurrence of PTH after TXA, saline or return to the OR. \| \| \| \| Exploratory Objectives: \| \| Establish pain and anxiety inventory at 7 +/- 1 from randomization. \| | | |  |
| **Endpoints:** | \| Primary Endpoint: \| Enrollment after one year, % of nebulization administered to patient's enrolled, and collection of two blood samples from each participant. Adverse events will be assessed at 30 +/- 5 days from randomization. \| \| --- \| --- \| \| Secondary Endpoints: \| Collection of surveys on ENT decision to go to the OR, electronic health record data on blood transfusions and estimated blood loss, and if PTH recurrences within seven days of randomization. \| \| Exploratory Endpoints: \| Age-appropriate pain and anxiety states at 7 +/- 1 days after randomization. \| | | | |
| **Study Population:** | Children aged 2 to 17 years old that present with post-tonsillectomy hemorrhage (PTH), ENT clinicians, parents of the children enrolled and missed eligible. | |  |  |
| **Phase:** | 2 | |  |  |
| **Description of Sites/Facilities Enrolling Participants:** | The University of Texas Health Science Center/University Health System for single site study then will expand to University of California at Davis, and Hasbro Children Hospital/Brown University School of Medicine | |  |  |
| **Description of Study Intervention:** | After randomization, children will receive three TXA nebulization doses (500 mg each) or three saline nebulized doses (placebo). | |  |  |
| **Study Duration:** | Study 1: 12 months, Study 2: 12 to 18 months | |  |  |
| **Participant Duration:** | Thirty days after randomization | |  |  |

## 1.2 Schema


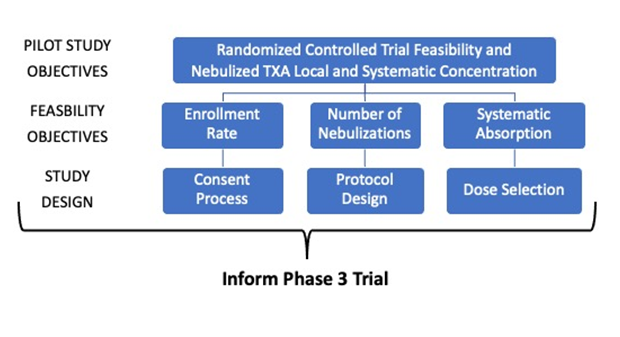


**Figure 1: Schema of TEAPOT Pilot**

Our overall study objective is to reduce the need for surgical control of post-tonsillectomy hemorrhage (PTH). Our primary objective for this pilot trial is enroll at least 0.6 patients/site/month and deliver at least two of three nebulization of TXA. The schema of our pilot trial will provide important data to improve the design of a larger Phase 3 trial.  Feasibility goals of the pilot trial include assessment of the consent process, the number of nebulization delivered to enrolled patients, and determine local and systematic concentrations of nebulized tranexamic acid (TXA) using a verified pulmonary physiologically based pharmacokinetic model. Data from the pilot trial will then help to inform a Phase 3 study design with an optimal consent process, pragmatic protocol, and dose selection of nebulized TXA to reduce the need for surgical control of PTH.

## 1.3 Schedule of Activities (SoA)

| **Procedure** | **Day 0-1***  **Enrollment** | **Day 7 +/- 1** | **Day 30 +/- 5 End of Study** |
| --- | --- | --- | --- |
| Screening and eligibility | X |  |  |
| Medical history | X |  |  |
| Consent | X |  |  |
| Demographics/baseline information | X |  |  |
| Concomitant medication review | X | X |  |
| ED/Hospital admission data collection** | X | X |  |
| Randomize to “use next box” | X |  |  |
| Administer study nebulization 1 | X |  |  |
| Administer study nebulization 2 | X |  |  |
| Administer study nebulization 3 | X |  |  |
| Surgical hemorrhage control (Y/N) | X | X |  |
| 1st TXA Level (last neb up to 60 mins) | X |  |  |
| 2nd TXA Level (60 mins to 8 hours) *** | X |  |  |
| ENT OR decision survey | X |  |  |
| Process, freeze, store, and ship samples | X |  |  |
| Age-appropriate Pain Scale |  | X |  |
| Parents and Children (Promis Anxiety Scale 8a) |  | X |  |
| Adverse and serious adverse events | X | X | X |

*Randomization to 24 hours

**Should include the following, if collected per standard of care: vital signs (including height and weight), physical exam, ENT upper airway exam, lab data collection (coagulation labs, other routine lab tests), OR use, ENT exam & findings as relating to OR use (e.g., photo of tonsillar bed), blood product usage, final hospital disposition. At seven days, further, evaluate if participants had been readmitted for bleeding after an intervention.

***Time points must be separated by 60 to 90 minutes.

# 2 Introduction

## 2.1 Study Rationale

Tonsillectomy is the second most performed surgical procedure on children in the United States, with more than 530,000 performed annually^1^. Four to five percent of children return to emergency departments (ED) for post-tonsillectomy hemorrhage (PTH)^2^ . This usually results in hospital readmission, blood transfusions, and/or surgical interventions^1,3^. To control PTH, more than 75% of pediatric otolaryngologists choose to go to the operating room for examination under general anesthesia and cauterize the bleeding source^4^. Non-operative management of PTH is challenging because visualization and application of direct pressure will obstruct breathing. Interventions such as gargles, silver nitrate, or topical hemostatic agents do not decrease the need for surgical hemostasis^5^. Development of a topical hemostatic agent for PTH remains challenging because of the extensive tonsillar vascular supply, constant exposure to saliva and bacteria, and shear forces from swallowing and coughing that detach the material from the tonsillar surface^6^.

The ideal treatment for PTH would be a drug that is effective, safe, and inexpensive. Tranexamic acid (TXA) is an antifibrinolytic drug, that competitively inhibits plasmin, part of the body’s natural process to degrade clots (Figure 3). Intravenous and topical TXA reduces blood loss for various types of pediatric surgery, without an increase in adverse events^7,8^. Nebulized TXA has also been shown to reduce bleeding in children with hemoptysis or pulmonary hemorrage^9^. In addition, a case report has documented that single dose of nebulized TXA given to a child with PTH may prevent the need for surgical hemostatic control^10^.  To confirm and extend these results, we completed a preliminary study evaluating nebulized TXA in 58 children with PTH. Three consecutive doses of nebulized TXA were given to 14 children at 500 mg per dose for children >25 kg and 250 mg per dose for children <25 kg. Our single-arm study demonstrated that nebulized TXA significantly decreased the surgical control of PTH by 44% compared to historical controls. There was no associated increase in adverse events or delays to surgery.


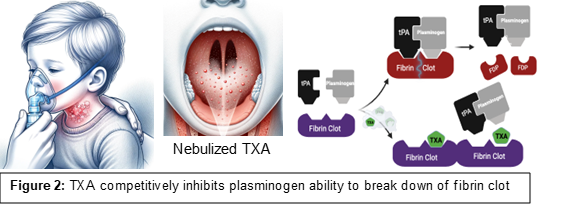


**Figure 2. Proposed mechanism of action of nebulized tranexamic acid (TXA) in post-tonsillectomy hemorrhage.** The left panel illustrates nebulized TXA delivery to the oropharynx. The center panel depicts the tonsillar fossa following tonsillectomy. The right panel illustrates the proposed antifibrinolytic mechanism whereby TXA competitively inhibits plasminogen activation and reduces fibrin clot degradation. The conceptual illustrations were generated using ChatGPT (OpenAI) based on author-developed scientific content and were subsequently reviewed, edited, and approved by the authors.

Children with PTH typically do not receive hemostatic interventions in the ED while awaiting the otolaryngologist’s arrival. In addition, many children with PTH present to hospitals that do not have immediate access to pediatric otolaryngology and thus require transport to tertiary hospitals. Nebulized TXA has the potential to be an easy, efficient, and safe ED intervention to stabilize potentially life-threatening bleeding from PTH and decrease the need for surgical interventions. Nebulized TXA likely has less side effects because the effect is topical, and little should be absorbed systemically. However, there are no studies that determine the local systematic absorption. Using known data from other studies and small number of systematic samples we can construct and verify a model that will determine the local and systematic absorption of nebulized TXA.

## 2.2 Background

The national incidence of PTH ranges from 2.6% to 10% and presentation varies from mildly darkened oral secretions to active hemoptysis^11,12^. However, the incidence may be under-reported, because most of these studies are retrospective with varying criteria for what constitutes a PTH. Additionally, ED studies report high rates of re-bleeding in patients that previously presented with a normal exam or stable clot^5^. Current non-operative PTH interventions, including hemostatic drugs, blood products, or topical agents have failed to prevent the need to return to the operating room and its associated risks^4^. Our first retrospective cohort document that three doses of nebulized tranexamic acid delivered for PTH appear to be safe and beneficial in a 44% reduction in the need for an operation to re-establish hemostasis^13^. Despite earlier reports that intravenous TXA increases the seizure risk in pediatric patients, no reports document that topical or local delivery of TXA causes adverse reactions^14^.

Non-operative control of airway and upper GI tract bleeding is of keen interest to pediatric surgeons, emergency, and intensive care unit health care providers. There is clear evidence that general anesthesia in children increases the risk of neurotoxicity, suggesting a need to avoid surgical management of PTH when safely possible^15^. Therefore, proven interventions that reduce operating room exposure would reduce morbidity and mortality. Systemic administration of TXA achieves hemostasis both intra- and post-operatively in orthopedic, dental, cardiovascular, and trauma literature^16^. However, systematic reviews document that intravenous TXA may not be effective when administered prophylactically or perioperatively to prevent PTH^17^. Additionally, large retrospective studies document that intravenous TXA can have significant delayed-onset adverse events including an increase in seizures, thromboembolic events, renal insufficiency, and changes in vision^14^.

Systemic administration of TXA achieves hemostasis both intra- and post-operatively in orthopedic, dental, cardiovascular, and trauma literature (19, 20). Systematic reviews document that intravenous TXA may be effective when administered prophylactically or perioperatively to prevent primary PTH but not secondary PTH (21). Additionally, large retrospective studies document that high-dose intravenous TXA can have significant delayed-onset adverse events, including seizures, thromboembolism, renal insufficiency, and changes in vision (17). Given the potential risks associated with systemic TXA administration, oral inhalation of TXA may have fewer side effects and be more effective. Studies document that inhaled drug formulations compared to intravenous increase airway and upper GI tract tissue exposure (22). Schwarz *et al.* was the first to report a case of a three-year-old boy to receive nebulized TXA who presented on postoperative day three with a PTH (23). The patient did return to the operating room, but the bleeding had stopped on arrival thus no operative intervention was needed. In 2021, we published a retrospective cohort documenting that three doses of nebulized tranexamic acid delivered for PTH in children appear to be safe and beneficial. This study documented a 44% reduction in the need for a second operation (24). Compared to earlier reports and studies, nebulized TXA is the first non-operative intervention that successfully decreased the need for an invasive procedure to control upper airway bleeding in children with PTH. This study was supported with a larger retrospective study of 83 patients treated, documenting a significant decrease in the need for operative control and repeat bleeding (25). Shin *et al* reported that TXA-treated patients had a decreased rate of operating room intervention of 36.1% versus 60.2% (p<0.0001) and a rate of repeat bleeding of 4.9% versus 14.2% (p<0.02). The odds ratio for operating room intervention with TXA treatment was 0.37 (95% CI 0.22, 0.63). There were no adverse effects identified with an average follow-up time of 586 days.

Nebulization of TXA is not a common route of administration compared to oral or intravenous delivery. Inhaled or intranasal medication can be rapidly absorbed across the large surface area of the respiratory tract epithelium. Some drugs absorbed into the pulmonary circulation enter directly into the systemic circulation via the pulmonary vein, possibly bypassing first-pass metabolism that occurs with oral drugs. Previous research into the site of action for inhaled medications has been limited to animal studies because biopsy of the entry site would be dangerous to the subject. However, to choose the optimal dose it is important for investigators to understand the level of absorption at the topical and systematic level. Typical pharmacokinetics of TXA cannot be done in this study, however the concentrations can be estimated by verifying physiologically based pharmacokinetic (PBPK) model designed to predict the change in pharmacokinetics that occur as the inhaled drug cross the respiratory epithelium. A pulmonary PBPK model begins with extracting from the literature all known pharmacokinetic data on the inhaled drug and the known properties for absorption and metabolism across the respiratory epithelium. Fortunately, several pharmacokinetic studies using healthy adults or animals have been performed with TXA. Additionally, several pediatric and inhaled pharmacokinetic studies are available. Utilizing this data, we can build a PBPK model to predict the level of topical and systematic absorption. The main advantage of this method is to understand how an inhaled drug is absorbed and metabolized in the respiratory epithelium with the fewest number of systematic samples. Instead of collecting a wide range of samples from different ages and doses, we only need a few samples to verify that the parameters used to build the model were correct. This technique has been used in multiple studies to predict the dosing response in various populations or using different routes of administration.

## 2.3 Risk/Benefit Assessment

### 2.3.1 Known Potential Risks

The known potential risk to the study will be minimized to include three categories:

A. Immediate Risk would include continued blood loss leading to worsening hemodynamic instability or airway loss. However, the study will not interfere with routine post-tonsillectomy hemorrhage care, therefore the risk of uncontrolled hemorrhage will be minimized.

B. Long Term Risk: The major long-term risk is from study design as a pilot study may not detect safety outcomes due to the small sample size. Another possible long-term risk is from toxicity from tranexamic acid organized into three categories:

1. Common but not harmful: headache, abdominal pain, oral, back, or muscle pain, or sinus congestion

2. Rare and serious: severe depression, allergic reaction, vision changes, seizures, thrombotic events, or ureteral obstruction

3. Rare but important or life-threatening: allergic skin reaction, anaphylaxis, cerebral thrombosis, achromatopsia, conjunctivitis (ligneous), deep vein thrombosis, diarrhea, dizziness, hypersensitivity reaction, hypotension (with rapid IV injection), nausea, pulmonary embolism, renal cortical necrosis, retinal artery occlusion, retinal vein occlusion, seizure, ureteral obstruction, visual disturbance, or vomiting.

C. Alternative Procedures: Current literature does not contain an established and effective non-invasive intervention for post-tonsillectomy hemorrhage. Non-operative management for active PTH includes conventional techniques such as direct pressure, clot suction, silver nitrate, and thrombin powder^4^.  The American Academy of Otolaryngology Surgery guidelines does not recommend any of these therapies because none have decreased the need for reoperation^18^. A double-blind randomized trial examined intravenous tranexamic acid delivered during pediatric adenoid-tonsillectomy, this study documented no decrease in perioperative blood loss or incidence of secondary bleeding. After this study, a meta-analysis was performed documenting a significant decrease in blood loss but did not find any change in post-tonsillectomy hemorrhage^19^.

### 2.3.2 Known Potential Benefits

The study's primary outcome is the need to go back to the operating room for hemostatic control within 24 hours from randomization. Although repeat surgery can restore hemostasis for post-tonsillectomy hemorrhage, it is not a guarantee of success. One small study of nonactive bleeding patients documented that the rate of rebleeding was 26/203 (12.8%) after inpatient observation and 3/21 (14.3%) after an operative intervention^5^. Our review of studies has revealed a modestly increased risk of adverse neurodevelopmental outcomes in children exposed to a single episode of general anesthesia and compounds with multiple exposures^15^.  Therefore, a reduction in exposure to a second surgery involving cauterization, anesthesia, and anxiety would significantly benefit children with post-tonsillectomy hemorrhage. Moreover, research into non-operative interventions will help future children who suffer from aerodigestive bleeding.  The benefits can be further classified into two groups:

A. **Immediate Potential Benefits**

1. Prevention of the need to return to the operating room for cauterization

2. Decrease in acute pain and anxiety in avoiding a procedure.

B. **Long-term benefits**

1. Reduction in anesthesia exposure may improve neurodevelopmental outcomes.
2. Avoidance of secondary procedure may improve early return to normal activities and improved quality of life in terms of benefits of the initial tonsillectomy including reduction of tonsillitis and sleep apnea symptoms.
3. The pilot study will inform an improved study design for a larger trial.
4. The pilot study will verify a PBPK model that will provide useful information on dosing and absorption of the nebulized TXA.

### 2.3.3 Assessment of Potential Risks and Benefits

Children with PTH typically do not receive hemostatic interventions in the ED while awaiting the otolaryngologist’s arrival. Also, many children with PTH present to hospitals that do not have immediate access to pediatric otolaryngology and thus require transport to tertiary hospitals. Nebulized TXA has the potential to be an easy, efficient, and safe ED intervention to stabilize potentially life-threatening bleeding from PTH and decrease the need for surgical intervention. Pilot trials in children designed with full-scale procedures help assess the feasibility and safety of large multi-center phase 3 trials. A smaller pilot trial can give go/no go information to progress to a larger phase 3 trial while minimizing the risks to the population. Additionally, starting with three doses of TXA may reduce the need for re-operation, further increasing the benefits of the study.  Determining the local (indirect estimate) and systematic (direct measurement) concentrations of nebulized TXA will help to choose an optimal dose for the larger trial. PTH is the leading cause of mortality in the second most often performed surgical procedure in children. Developing an effective, non-invasive, low-risk solution would benefit public health by addressing the life-threatening complications PTH in children.  

# 3 Objectives and Endpoints

| OBJECTIVES | ENDPOINTS | JUSTIFICATION FOR ENDPOINTS |
| --- | --- | --- |
| Primary | | |
| Evaluate the feasibility to enroll 0.6 patients/site/month  Nebulize at least two of three doses of TXA to children with PTH.  Determine the local and systematic concentrations of nebulized TXA in children  Determine incidence of adverse and serious adverse events | Enrollment after one year and % of nebulization’s. Go/no-go threshold is 0.6 patients per site per month for a total of 22 patients. Time and success to obtain consent will establish need for exception for informed consent (EFIC).    The goal is >90% of patients will receive at least two of three planned nebulization of TXA.  Collection of two blood samples from each participant. This will verify a pulmonary physiological-based PK model (PBPK) (i.e., nasal cavity, pharynx, and lung) that indirectly predicts the oropharyngeal and systematic concentration of nebulized TXA.  A phone call or email will be placed to participants at 30 +/- 5 days. | The overall incidence of PTH is 5%, yet numbers per site may be very different. Assessment and consent will optimize site selection, recruitment process, and the plan the main trial.    The establishment of a pragmatic protocol design will help design a large trial.  Limited data on nebulized TXA systematic absorption. Topical PK studies of TXA document a significant reduction in systematic levels but the same hemostasis effect.  Safety Evaluation as recommended by the FDA. |
| Secondary | | |
| Determine the number and rationale for PTH patients to return to the OR; and    Determine the number of blood transfusions and estimated blood loss and      Determine the recurrence of PTH after TXA, saline, or return to the OR. | Collection number participants and surveys on ENT surgeons’ decision to go to the OR    Collection of electronic health record data on number of blood transfusions and estimated blood loss    Survey families to determine PTH recurred seven days from randomization. | To evaluate if return to the OR can be an outcome for the main trial.    To evaluate if TXA decreased severity of PTH bleeding.      To evaluate if TXA or return to OR prevented PTH recurrence. |
| Tertiary/Exploratory | | |
| Establish pain and anxiety after receiving study intervention. | Age-appropriate pain and anxiety state (PROMIS) at 7 +/- 1 days after randomization. | Surgical procedures provoke significant parental and child anxiety. Reduction in parent and child anxiety is a strong patient-centered outcome of quality care. |

# 4 Study Design

## 4.1 Overall Design

Our overall hypothesis for the phase 3 trial is that nebulized TXA will decrease the severity of bleeding and the need for repeat surgical intervention for PTH. To test this hypothesis, we first propose to conduct a multi-center, randomized controlled, blinded pilot trial (phase 2) to assess the feasibility, safety, and the local and systematic absorption of nebulized TXA in children with PTH. Safety and pharmacokinetic data are well known for topical and intravenous administration of TXA but there is limited data on nebulized delivery of TXA. Participants will be randomized at the time of the consent in the emergency department. Earlier PECARN studies experience suggest that pre-consenting patients in surgical clinic will not improve participant enrollment or completion. Children 2 to 17 years old presenting to the ED with PTH will be randomized in a 1:1 ratio to three doses TXA or placebo (saline).  All interventions will be blinded by the investigator, treating clinical care team, and research staff. Only the pharmacist and biostatistician will know the randomization schedule. We will not unblind patients as there is no reversal agent. Clinical teams will be asked to assume the patient received TXA. Patients will be followed during their hospital course and outcomes will be evaluated at seven +/- 1 days after randomization. As this is a pilot study, no stratification or interim analysis will be performed.


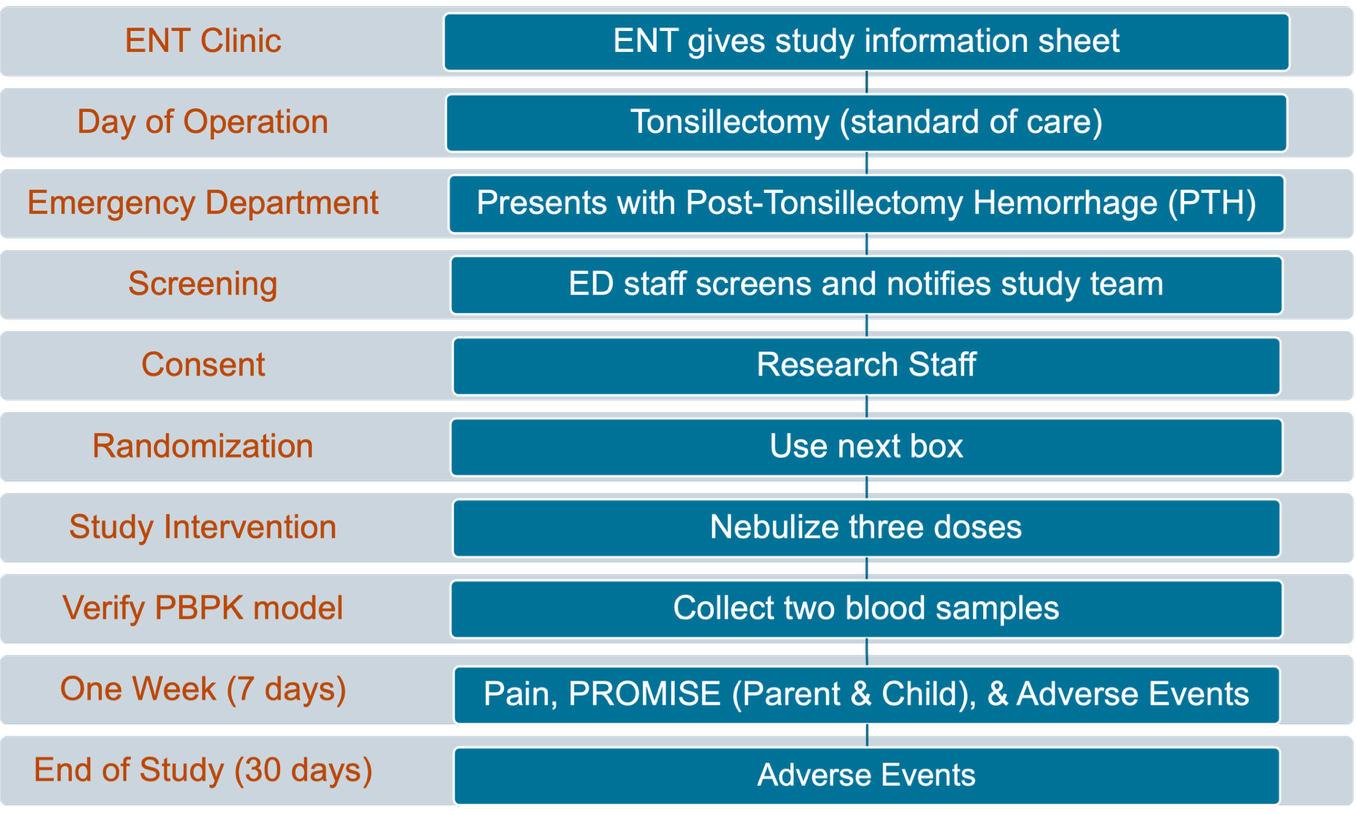


**Figure 3: Study Protocol Flow**

## 4.2 Scientific Rationale for Study Design

In 2018, a case report documented that a single dose of nebulized tranexamic acid (TXA) prevented operative management in a three-year-old with PTH^10^. A subsequent randomized controlled trial in adults documented a decrease in blood loss and secondary hemorrhage using nebulized TXA for hemoptysis^20^. The current use of antifibrinolytics (e.g., TXA or e-aminocaproic acid) is rarely used to treat PTH. Therefore, there is an essential need to test the efficacy of nebulized TXA to decrease the severity of PTH. Our retrospective cohort study documented that nebulized TXA may be effective in decreasing the need for operative intervention^13^. However, retrospective studies have significant bias indicating the need for a multi-center, randomized, double-blinded, controlled clinical trial. As we do not have an established alternative drug the standard is to test the drug vs. a placebo such as nebulized normal saline. The power analysis and 1:1 study design reduces bias and confounders for the evaluation of study outcomes. Even if the patient presents without active bleeding, it is possible that nebulized TXA has the potential to stabilize the clot to prevent future bleeding.

A verified pulmonary PBPK model can set the dose of the phase 3 trial. A pulmonary PBPK model incorporates all known intravenous, oral, or local TXA administration PK and pulmonary physiology data. This model only needs verification by small number of systematic samples, compared to traditional pharmacokinetic studies that require multiple samples using different doses and ages of patient. A verified model can then be use predict the drug for the phase 3 trial with the lowest systematic absorption, which will minimize side effects.

## 4.3 Justification for Dose

We conducted a study evaluating nebulized TXA in 58 children with PTH^13^. Three consecutive doses of nebulized TXA were given to each child, 500 mg per dose for children >25 kg and 250 mg per dose for children <25 kg. These doses were picked from the earlier literature ^9,21^ that documented success in reducing hemoptysis. Our single-arm study demonstrated that nebulized TXA significantly decreased the need for surgical management of PTH by 44% compared to historical controls. There was no associated increase in adverse events or delays in care to patients who required a surgical intervention. This study found that some children responded after one dose, but most children responded after two doses. For the pilot trial, we propose to administer three doses of 500 mg (total 1500 mg) of nebulized TXA to each child enrolled regardless of their weight. This was further confirmed by a retrospective study that delivered 500 mg/dL to several children less than 7 years of age(3). We considered dose choice based on the need to balance the amount of TXA with a goal to decrease the severity of PTH. Using TXA improves our safety because it is an approved medication that has a minimal and known side effect profile.

We chose a total dose of 1500 mg because it does not exceed the maximum intravenous dose approved for the average weight of a four-year-old. Neurological and cardiac surgical procedures have used over 100 mg/kg intravenous loading dose of TXA and then 10 mg/kg/hour drip with minimal side effect^22,23^. We hope that nebulization to deliver TXA will result in a lower systematic peak of TXA and yet because it is focused on the source of the bleed it remains effective. Several studies have documented that peak TXA concentration is much reduced with topical versus intravenous administration^2425^. Lastly, the potential benefits of a higher dose of TXA is significantly less risk than risk of neurocognitive injuries documented by repeat exposures to anesthesia and surgery. In summary, the investigators chose a fixed dose based on the two points: 1) Nebulized TXA is expected to have very little systemic uptake, and we wish to maximize safe effect, and 2) Prior surgical studies have not shown an increased risk of AEs with intravenous TXA compared to placebo.

## 4.4 End of Study Definition

Study drug or placebo will also be discontinued if a severe anaphylactic reaction during or after administration.   All participants will be followed adverse events, need for surgical control of PTH, and patient-related outcomes for up 7-30 days from receipt of study drug.

# 5 Study Population

## 5.1 Inclusion Criteria

1. Received a tonsillectomy

2. Presents to the ED with secondary* post-tonsillectomy hemorrhage

3. Children between age of 2 to 17 years of age (i.e., before their 18^th^ birthday)

*Secondary post-tonsillectomy hemorrhage is defined as greater than 24 hours from their primary tonsillectomy operation (arrival in recovery/PACU).

## 5.2 Exclusion Criteria

1. Known and documented bleeding or clotting disorder

2. Known pregnancy

3. The patient is a ward of the state

4. Patients with known hypersensitivity or allergic response to tranexamic acid

5. Parents or guardians that cannot communicate in English or Spanish.

6. Intubation prior to enrollment

7. Previously enrolled patients

## 5.3 Lifestyle Considerations

Prior to performing a tonsillectomy, otolaryngologists will routinely screen all females who are at childbearing age for pregnancy. Thus, significantly decreasing the risk of enrolling an unsuspecting female who is pregnant. Furthermore, the risk of tranexamic use in pregnant females is minimal and unlikely because the use of oral tranexamic acid for the long-term prophylaxis of bleeding in pregnant females is well documented to be safe and effective^21^. There is no evidence from animal studies that tranexamic acid has any teratogenic effect or risk to the fetus (Category B). Reproductive studies have been performed in mice, rats, and rabbits and have no evidence of impaired fertility or risk to the fetus. Lastly, the investigators are concerned that imposing this exclusion criterion will reduce enrollment.

It is also possible that non-steroid anti-inflammatory drugs would influence the bleeding outcomes of the study participants. However, a 2013 updated systematic review and meta-analysis of 36 randomized controlled trials documented no significant differences in rates of bleeding or reoperation in patients receiving NSAIDS post-tonsillectomy^2^.

## 5.4 Screen Failures

**Missed eligible** patients are those who meet inclusion and exclusion criteria but are not enrolled. We will ask for a waiver of consent to review and collect emergency department records at each site (up to 100 patient's) during the study period to determine if patients met eligibility criteria but were not enrolled into the study. This can be due to misunderstanding, misapplying, not meeting the exclusion criteria or other factors.

**Screen failures** are defined as participants that consent to take part in the clinical trial but are later found to be ineligible.  A minimal set of screen failure information must ensure transparent reporting of screen failure participants to meet the Consolidated Standards of Reporting Trials (CONSORT) publishing requirements and to respond to queries from regulatory authorities. Minimal information includes demography, screen failure details, eligibility criteria, and any serious adverse event (SAE). Individuals who do not meet the criteria for participation in this trial (screen failure) may be re-screened. Rescreened participants will be assigned a new ID number as it is a new encounter.

## 5.5 Strategies for Recruitment and Retention

The pilot study aims to test the feasibility of enrolling 0.6 patients per site per month. The specific goal for this pilot trial is to enroll 12 patients (6 treatment groups & 6 control group) at the University of Texas Health Science Center (UTHSCSA)/University Hospital in San Antonio, TX. The participants will be children who present to the emergency department (ED) with secondary posttonsillectomy hemorrhage (PTH) occurring 24 hours or more after their tonsillectomy. PTH patients will be screened and approached in the ED. Participants will be followed as outpatients for one week post-randomization. Due to the short follow-up period, we do not expect a significant loss of participants. However, text, phone, and e-mail information will be obtained from each participant to give the study team multiple options for gathering data on secondary and tertiary objectives. In this pilot analysis, we will offer participants an incentive to complete all study procedures. We will also closely monitor study completion to determine if participant incentives are necessary for the main trial. This pilot study will help assess the feasibility of enrollment rates and the effectiveness of follow up methods, which will inform the design and implementation of the larger main trial.

The investigators will enroll at the University of Texas Health Science Center (UTHSCSA)/University Hospital in San Antonio, TX for the single site and the multi-site study, the University of California at Davis Medical Center in Davis, CA, and Hasbro Children’s Hospital in Providence, RI. Each of the ENT and ED colleagues at each site have provided the incidence of PTH, percentage that return to the OR, and the monthly ED visit rate. Previous studies document that PTH primarily presents at night, therefore we arrange for 24/7 coverage at each site.

| **Site** | **Annual PTH Incidence** | **Going to OR** | **Monthly ED Visits** |
| --- | --- | --- | --- |
| San Antonio, TX | 5.4% | 73% | 1.60 |
| Davis, CA | 3.2% | 70% | 0.63 |
| Providence, RI | 2.8% | 63% | 2.80 |

5.6 COSTS

The patient or their health insurance provider will bear the responsibility for the expenses associated with treatments and procedures that would be administered regardless of the patient's participation in this study, such as subsequent surgeries and hospital admissions. Please note that certain costs may not be covered by insurance providers (e.g., approved drugs used off-label). If the patient's insurance does not cover these treatments or procedures, the patient will be accountable for the associated expenses.

"The sponsor will supply the study drug/device at no cost to patients throughout the duration of this study.

5.7. COMPENSATION

Payment method: participants will receive compensation via an hsc debit card.

Compensation details:

- Visit 1: day 0-1
  - Type: one-time
  - Activity: initial visit
  - Amount: $50 (usd)
- Visit 2: day 7
  - Type: one-time
  - Activity: second follow-up visit
  - Amount: $25 (usd)
- Visit 3: day 30
  - Type: one-time
  - Activity: final visit
  - Amount: $25 (usd)

Timing: compensation will be provided immediately upon completion of each visit.

Minimizing coercion: the compensation structure has been carefully designed to avoid coercion or undue influence. The amounts offered are reasonable and commensurate with the time and effort required for each visit, ensuring that participants are not unduly influenced to participate.

Pediatric participants: compensation will be provided to the parent or legal guardian of the minor participant.

Prorated compensation: if a participant withdraws from the study before completion or if there are multiple research activities, compensation will be prorated accordingly based on the completed activities.

Reimbursement: participants may be eligible for reimbursement of travel expenses such as hotel accommodation or mileage. Reimbursement will be provided upon submission of receipts, and the reimbursement process will be explained during the informed consent process.

Escort reimbursement: there may be reimbursement available for escorts who accompany participants to study visits. Details regarding escort reimbursement will be provided upon request and will be subject to the study's reimbursement policy.

# 6 Study Intervention

## 6.1 Study Intervention(s) Administration

### 6.1.1 Study Intervention Description

The intervention will not interfere with or supersede standard ED care for post-tonsillectomy hemorrhage. After determination of eligibility and obtaining informed consent, patients will be randomized to receive nebulized TXA or saline. The only change to ED PTH management is the addition of nebulized study drug. This randomized pilot trial will thus evaluate the feasibility to enroll patients into a trial that will determine the efficacy of nebulized TXA (investigational drug) compared to nebulized normal saline (placebo) to prevent repeat surgical intervention.

### 6.1.2 Dosing and Administration

The investigational product, TXA 100 mg/mL, is approved in a pediatric population for treatment of hemorrhage post-tooth extraction, and for short-term treatment of hemophilia. For this study, nebulized TXA will be administered in a double-blinded randomized protocol to children who are 2 to 17 years of age, using nebulized saline as a placebo. The study interventions should not interfere with standard of care. is not needed, the clinical nurses or respiratory therapists can administer a study medication per protocol, after receiving protocol training.

## 6.2 Preparation/Handling/Storage/Accountability

### 6.2.1 Acquisition and accountability

The study drug will be purchased or supplied by American Regent (generic version of the injectable 100 mg/mL) in bulk as 0517-0960-10 × 10 mL single dose vial. The placebo (0.9% sodium chloride, NDC 0517-2810-25 x 10 mL single dose vial) will be purchased from a similar manufacturer and then repackaged by a Hybrid Pharma, 1015 W. Newport Center Drive, Suite 106A, Deerfield Beach, Fl-33442, [hp@hybridpharma.com](mailto:hp@hybridpharma.com), Ph: 954 708 2771, Fax: 954 708 2993 into identical vials that TXA originally is packaged in. Study drugs will be received by a hospital investigational pharmacist, who will prepare “Use Next Boxes” for the ED.

The nebulizer mask used to deliver the TXA will be provided with the study drug as the PARI LC® D Disposable Nebulizer (PARI Respiratory Equipment, Inc., Midlothian, VA) or a similar jet nebulizer based on supplies.

### 6.2.2 Formulation, Appearance, Packaging, and Labeling

Study drug and saline will come in similar vials, as described above, that can be blinded by placing a label over the original packaging.   The pharmacist will blind (please see example in Figure 4) two vials of study drug or placebo assigned per randomization schedule. Then they will be placed in the "Use Next Box" for the randomization and dosing process.


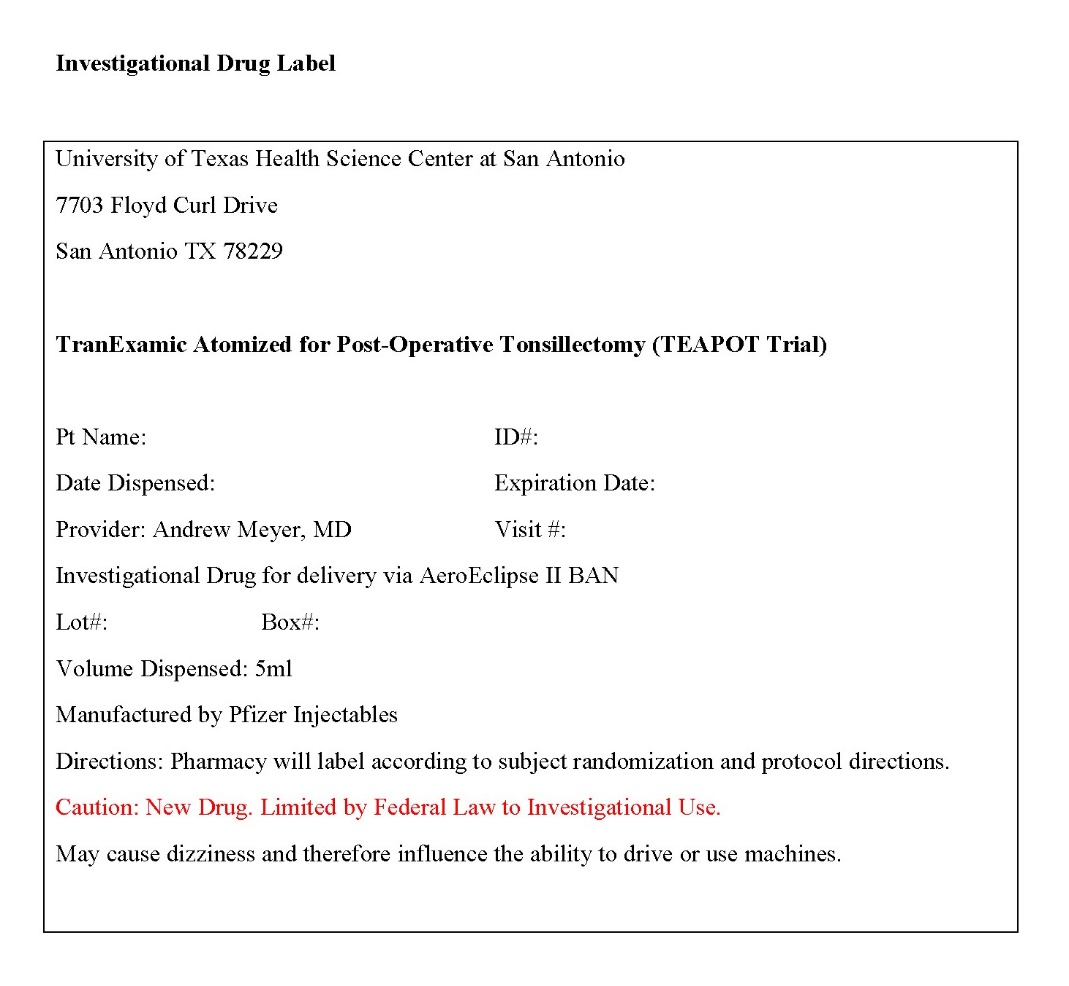


**Figure 4: Study Drug Investigational Drug Labelling.**

### 6.2.3 Product Storage and Stability

For TXA and saline: Store at 20°C-25°C (68°F-77°F); excursions permitted from (59°F-86°F) [see USP Controlled Room Temperature].

### 6.2.4 Preparation

Upon enrollment into the study, clinical staff will load the study drug into a nebulizer well (PARI LC® D Disposable) and then deliver the TXA or placebo via a mask. Study drug will be nebulized using the hospital gas supply. All personnel except the investigational pharmacist and/or statistician will be blinded to the drug. The randomization schedule will be pre-determined by a central randomization process and prepared as "Use Next boxes" to decrease the need for the randomization process at enrollment.  "Use Next Boxes" will have three study drug doses (blinded by an investigational pharmacist), a study nebulizer, and instructions for study drug delivery.

## 6.3 Measures to Minimize Bias: Randomization and Blinding

Due to the narrow time window of study intervention, randomization must not delay treatment. To complete the randomization quickly, the study intervention will be preassigned using a central randomization process. Prior to enrollment at each site, a study drug box having two vials (1000 mg per vial) of blinded study drug with a numeric identification code corresponding to the treatment assignment will be designated as the “Use Next Box." All interventions will be blinded to the investigator, clinical care team, and research staff. Only the pharmacist and biostatistician will know the randomization schedule. Unblinding is not allowed. Clinicians will be instructed to assume that the patient has received TXA and treat accordingly.

## 6.4 Study Intervention Compliance

Every week of the study enrollment, study staff will review and complete data entry for patients screened or missed eligible patients. All relevant surgical and clinical data will be collected related to risk factors for PTH, pharmacokinetics, or receipt of study drug or placebo (please see the schedule of activities). Relevant data collection for enrolled PTH patients includes BMI, historical platelet counts, episodes of bleeding or clotting, insurance, ethnicity, surgical tonsillectomy technique performed, prescribed and over the counter medications, and other data as will be described in the manual of operations. Data collected on missed eligible patients, using a waiver of consent, will be age, sex, height, and weight if available, medications taken upon admission, insurance, and outcomes including surgery performed or not. For more details see the Manual of Operations. Data will be collected and managed using REDCap hosted at the University of Utah. REDCap (Research Electronic Data Capture) is a secure, web-based application designed to support data capture for research studies. Below is a table of associate responsibilities.

| **Procedure** | **Data Source** | **Responsible** |
| --- | --- | --- |
| Screening and eligibility | Electronic Health Record (EHR) | Research Staff* |
| Medical History | EHR or study staff | Clinical and Research Staff |
| Consent | E-consent, Virtual, or in Person | Research Staff |
| Demographics/baseline information | EHR or study staff | Clinical and Research Staff |
| Concomitant medication review | EHR | Clinical and Research Staff |
| ED/Hospital admission data collection | EHR | Clinical and Research Staff |
| Administer study nebulization 1 | EHR or study staff | Clinical and Research Staff |
| Administer study nebulization 2 | EHR or study staff | Clinical and Research Staff |
| Administer study nebulization 3 | EHR or study staff | Clinical and Research Staff |
| Surgical Hemorrhage Control? (Y/N) | EHR or study staff | Research Staff |
| 1st TXA Level (last neb up to 60 mins) | Blood Sample | Clinical and Research Staff |
| 2nd TXA Level (60 mins to 8 hours) | Blood Sample | Clinical and Research Staff |
| Process, Freeze, Store and Ship | Plasma Samples | Research Staff |
| ENT Surgical Decision Survey | ENT Surgeon | Research Staff |
| Age-Appropriate Pain Scale | EHR, Text, or Phone | Research Staff |
| Promis Anxiety Scale (Parent and Child) | EHR, Text, Phone, or Virtual | Research Staff |
| Adverse Events/UPIRSO | Text, Phone, Virtual | Research Staff |
| Serious Adverse Events | Text, Phone, Virtual | Research Staff |
| Missed Eligible | EHR | Research Staff |

* Research staff can refer to research coordinators, co-investigators, principal investigator, or other study team members who have been trained to be part of the study

## 6.5 Concomitant Therapy

TXA has very few if any drug metabolism interactions. Patients will be allowed to take over-the-counter medications for pain that affect bleeding (e.g., non-steroidal anti-inflammatory drug), birth control (estrogen-containing products), or retinol A. Records of all doses and frequency of these medications will be collected during the perioperative course through retrospective chart review.

### 6.5.1 Rescue Medicine

There is no rescue medication for the study drug (TXA or placebo). If there is an allergic reaction, the study drug will be stopped, and routine care for allergic reactions will be followed.

# 7 Study Intervention Discontinuation and Participant Discontinuation/Withdrawal

## 7.1 Discontinuation of Study Intervention

Study drug or placebo administration will be discontinued if the patient experiences any allergic or anaphylaxis reaction. If a severe anaphylactic reaction or severe vomiting occur during or after administration, clinical staff and study team should assume the patient received a study drug as there will be no unblinding procedures.

Discontinuation from study drugs does not mean discontinuation from the study, and remaining study procedures should be completed as indicated by the study protocol.  If a clinically significant finding is identified (including but not limited to changes from baseline) after enrollment, the investigator or qualified designee will determine if any change in participant management is needed. Any new clinically relevant finding will be reported as an adverse event (AE). The data to be collected at the time of study intervention discontinuation will include the following pharmacokinetic studies and clinical case report forms as indicated above.

## 7.2 Participant Discontinuation/Withdrawal from the Study

Participants are free to withdraw from participation in the study at any time upon request. A site investigator may discontinue or withdraw a participant from the study for the following reasons:

1. If any clinical adverse event (AE), laboratory abnormality, or other medical condition or situation occurs such that continued participation in the study would not be in the best interest of the participant.
2. Emergent intubation for respiratory failure.
3. If the participant meets an exclusion criterion (either newly developed or not previously recognized) that precludes further study participation.

The reason for participant discontinuation or withdrawal from the study will be recorded on the Adverse Event Case Report Form. Subjects who sign the informed consent form and are randomized but do not receive the study intervention may be replaced.  Subjects who sign the informed consent form, are randomized, and receive the study intervention, and later withdraw or are discontinued from the study, will no longer have data collected, but existing data collected prior to withdrawal will still be used. Subjects may be replaced as necessary depending about withdrawal.

## 7.3 Lost to Follow-Up

A participant will be considered lost to follow-up if we are unable to measure the secondary outcomes up to seven to thirty days from randomization including the need for surgical intervention (Y/N) or additional bleeding. At least 3 attempts will be made to follow up with the participants using contact information provided during the consent process.

# 8 Study Assessments and Procedures

## 8.1 Efficacy Assessments

The **primary feasibility outcomes** for the pilot trial are the enrollment rate (patients per site per month) and the proportion of patients receiving study drugs (at least 2 nebulized doses).  Safety is always an outcome, and all adverse events will be reviewed.

**The secondary outcome is** the ENT surgeon rationale, decision (Y/N) for the PTH patient to return to the OR, severity of bleeding with estimates of blood loss that include volume of hemorrhage recorded blood loss, and the number of blood transfusions. Additionally, we will determine the indirect local and systematic concentration of nebulized TXA by verifying our pulmonary physiologically based pharmacokinetic model. To verify the model, two lab samples will collect at opportunistic times either by finger stick or ad**. The study is not** powered for the secondary outcomes as this a pilot trial.

The **exploratory outcomes** will be age-appropriate pain scores and parent and child anxiety scales as measured by anxiety-state-trait scale (STAI). For children under eight years old their anxiety will be measured by STAI-C, which utilized pictures These will be exploratory as the pilot trial is not powered to determine the change in the outcomes.  Please see **Figure 7** for a timeline of all study procedures and outcomes.

## 8.2 Safety and Other Assessments

## 8.2.1 Safety OUTCOMES

Incidence of safety outcomes will be assessed at 1, 7 and 30 days after receipt of the study drug via a review of electronic health records and interviews. Specific, safety outcomes are thromboembolic disease: any venous or arterial thrombosis on standard diagnostic imaging post-randomization (including deep vein thrombosis, pulmonary embolism, sinus thrombosis, myocardial infarction, ischemic stroke) and seizures occurring within 24 hours (greater than 5 half-lives of TXA) of drugs documented by clinical or electroencephalogram.

## 8.2.2 HOSPITAL DATA COLLECTION and Patient-related OUTCOMES

**Electronic Health Record:** Data will be recorded for all enrolled patients to include age, sex, race and ethnicity, and chronic medical conditions. Upon presentation to the ED, we will also record vital signs and physical examination findings. We will collect baseline laboratory values from electronic health records that are routinely collected as the standard of care. Surgery-specific information will also be collected from electronic health records or charts including the type of tonsillectomy, description of photos of tonsillar bleeding, subject demographics (e.g., ethnicity), insurance, and clinical information. Moreover, a brief survey will be given to each attending ENT physician to document their rationale and concerns to go to the OR.

**Clinical Data Examples:** Should include the following, if collected per standard of care: vital signs (including height/weight), physical exam, upper airway and pulmonary exam, exam & findings as relating to OR use (e.g., photo of tonsillar bed), blood product usage, final hospital disposition. A recent height and weight of all patients should be collected from the electronic health record or from parents as body mass index (BMI) has been correlated with the severity of PTH.

Physician Survey: A post-operative survey will be distributed attending emergency and ENT physicians and staff that evaluate experience with patients PTH. The survey will be brief to have the physician detail the reasoning behind the decision to take the patient back to a second surgery or not. The remaining questions will be to describe the exam of the PTH in the operating room if performed. The surveys will be distributed electronically within 24 hours of randomization. The surveys will contain the following preamble “**Dr. Andrew Meyer, Associate Professor of Pediatrics at the University of Texas Health and Science Center, will be conducting a research study on Pediatric post-Operative Tonsillectomy hemorrhage. Participating is completely voluntary and participating or not participating in this research study will not affect an individual's academic or professional standing. Willing participants will be asked to complete this 10-minute survey that will be administered through a QR code or on paper. There will be no compensation for participating in this study. To protect your confidentiality, survey responses are anonymous, and no personally identifiable information will be asked. As an extra precaution, please do not write in information that could possibly identify you on the open-ended questions. If you have any questions, please reach out to Dr. Andrew Meyer at meyera@uthscsa.edu.**”

**Laboratory Data Examples** (collected if completed as the standard of care procedures): complete blood count, INR/Prothrombin time, activated partial thromboplastin time (aPTT), D-dimer, Thromboelastography, fibrinogen, and arterial or venous blood gas.

**Pharmacokinetic and Pharmacodynamic Testing:** Pharmacokinetics samples will be collected after completion of the last nebulized treatment received within sixty minutes. A second time point should then be collected after sixty minutes up to eight hours from last nebulization, separated from the previous time point by at least sixty to ninety minutes. The results from the TXA level measurements will be used for research purposes only and not be available to clinicians. The serum TXA levels will be used to develop a TXA PK model and determine the population variability. We will develop a base model to determine a best-fit compartmental model, distribution, and elimination kinetics. We will also use stochastic models to evaluate between-subject variability in PK parameters. Serum TXA concentrations will use 200 microliters of plasma measured by an ultra-high performance liquid chromatography tandem mass spectrometry method specifically developed for sensitive and precise analysis of low TXA concentrations at the U.S. Army Institute for Surgical Research. Moreover, we will use the remaining 200 microliters to measure pharmacodynamics effects of nebulized TXA in the plasma. ELISA can use minimal plasma volume (<300 μL) to run commercial kits measuring Plasmin-antiplasmin (PAP) complexes, D-dimer, Thrombin-antithrombin (TAT) complexes, Prothrombin fragment F1+2 (F1+2), and Plasminogen activator inhibitor-1 (PAI-1). Combination of pharmacokinetic and pharmacodynamic effects achieves the primary goal to determine drug systematic compared to local effects.

The protocol will adhere to the following guidelines when scheduling procedures to collect pharmacokinetic samples:

- Samples will be collected in EDTA 1 mL tubes (Fisher Scientific Catalog number 22-030-402). Within 2 hours of sample collection, tube will spin in a calibrated centrifuge. Please see attached centrifuge calibration standard of operating procedure.
- Centrifuge procedures consist of:
  - Place sample in centrifuge with appropriate weighted balance
  - Centrifuge sample at 2500 RPM for ten minutes at room temperature
  - Remove sample from centrifuge, using a pipette remove plasma above buffy coat and place into a clean Eppendorf tube (roughly 0.4-0.6 mL).
  - Place Eppendorf tube back in centrifuge with appropriate balance.
  - Centrifuge second time at 2500 RPM for ten minutes at room temperature.
  - Remove sample from centrifuge, using a pipette to remove 7/8^th^ of sample and aliquot equally into two labelled cryovials (roughly 0.2 mL). Each vial will be labelled with participant enrollment number, date and time of collection, and either “TEAPOT PK” or labeled “TEAPOT PD”.
  - Store samples in -70°C to -80°C freezer.
- All sample shipments will follow IATA and cold-chain management guidance.
- Specimens submitted frozen can be collected on any day but must be stored frozen and shipped to the U.S. Army Institute of Surgical Research on Monday through Thursday. If frozen specimens cannot be shipped immediately, they must be maintained in a -70°C to -80°C freezer per cold-chain-management procedures.
-
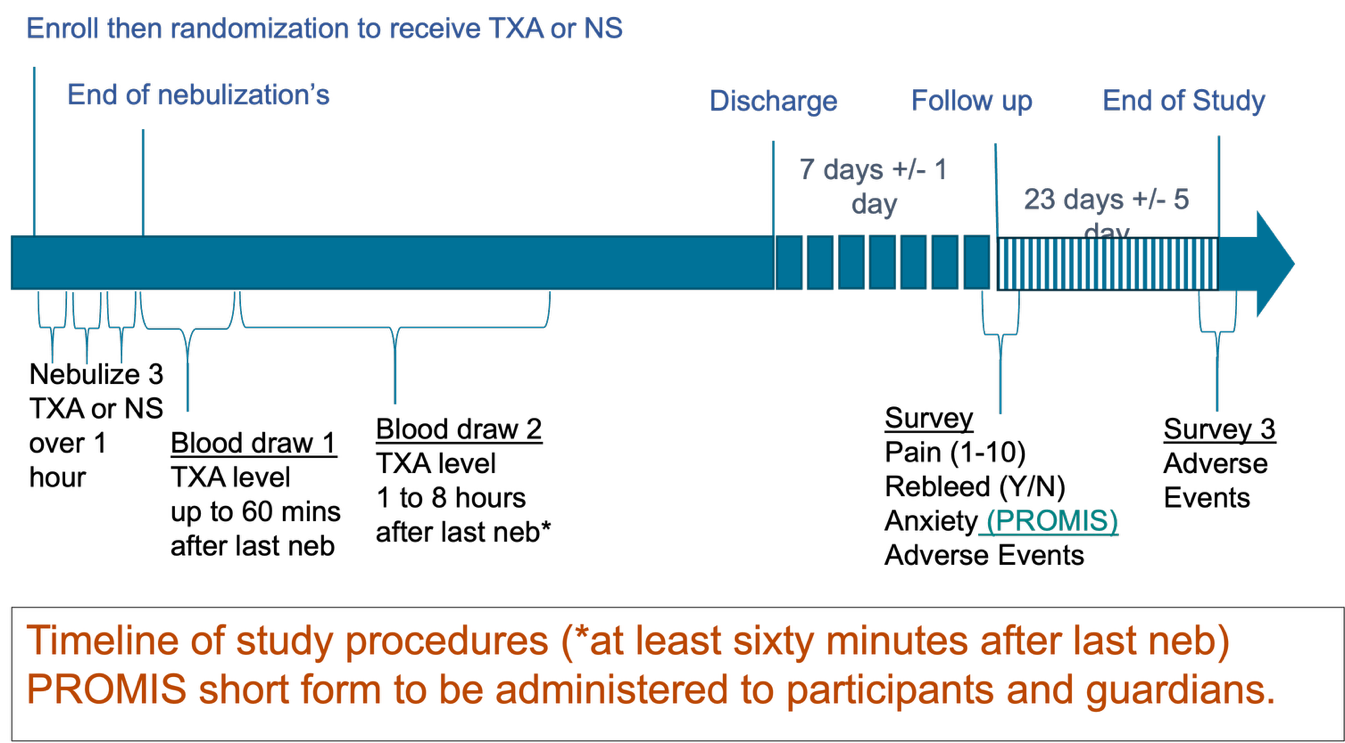
Fresh blood specimens may be collected, processed, frozen and shipped Monday through Thursday during normal business hours.

**Figure 5:** Timeline of Study Procedures (*at least six minutes after last neb) STAI is the State-Trait Anxiety Inventory to be administered to participants and guardians. *AEs will be collected through a phone call at 30 days +/- 5 days*

Coded specimens with dates will be sent to University of Utah and/or U.S. Army Institute of Surgical Research for pharmacokinetic analysis. Please see Figure 5 timelines of events.

**Blood product transfusion volume and Blood Loss:** Blood product transfusion volume will be measured at discharge or 24 hours (whichever comes first). This will include the volume of packed red blood cells, platelets, plasma, cryoprecipitate, or whole blood. Any mention of blood loss in electronic health records from emergency, anesthesiology, or surgeons’ notes will be recorded.

**Hospital Discharge Information:** Information will be collected after hospital discharge including otolaryngologist (ENT) notes and physical exam documentation, hospital discharge date and time, destination upon discharge, and mortality.

## 8.2.4 FOLLOW UP DATA COLLECTION

**The secondary outcome** (the need for return to the OR for surgical management of PTH) will be followed for up to seven days after randomization.

**Patient-related outcomes measures (PROM)** include pain (as measured by age-appropriate pain scale) and parental/patient anxiety (measured at 7 days after randomization).  The rationale for measuring parent and child anxiety and pain have been documented as measures that significantly predict overall parent satisfaction with perioperative care^28^. To assess patient and parent-centered outcomes of parental and child anxiety we will assess the following scores at day 7 +/- 1-day post-study drug randomization.

1. PROMIS Short Form v1.0 - Anxiety - 8a 31 May2019, PROMIS Early Childhood Parent Report SF v1.0 - Anxiety 8a - 4-1-2021, and Numeric Rating Scale for Anxiety (NRS-A)
2. Wong-Baker Age-appropriate Pain Score & FLACC Behavioral Assessment Scale

For discharged patients, parents will be contacted by a HIPAA compliant text messaging (e.g., TigerConnect App) or phone call for these assessments and communication. If the patient is still in the hospital, research staff may conduct these follow-ups in person.

Wong-Baker FACES® Pain Score:


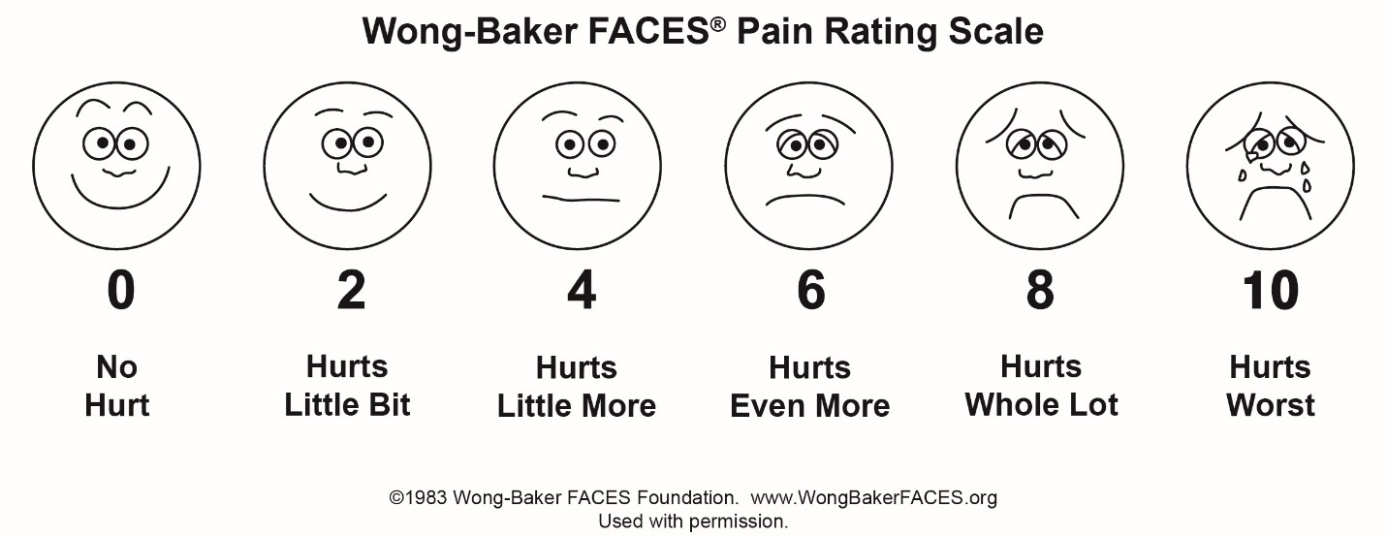


 FLACC Behavioral Pain Assessment Scale

**Parental and child state and trait anxiety**: To assess anxiety in both parents and pediatric participants, we will use validated Patient-Reported Outcomes Measurement Information System (PROMIS) instruments and a Numeric Rating Scale for Anxiety (NRS-A).

1. PROMIS Anxiety Measures

Older children, adolescents, and parents: Anxiety will be measured using the PROMIS Short Form v1.0 – Anxiety – 8a (31 May 2019). This is an 8-item self-report instrument designed to assess symptoms of fear, worry, hyperarousal, and somatic anxiety over the past 7 days. Items are scored on a 5-point Likert scale (Never to Always). Raw scores are converted to standardized T-scores (mean 50, SD 10), allowing comparison to the U.S. general population. Higher T-scores indicate greater anxiety severity.

Young children (early childhood): For children aged 4–7 years, anxiety will be assessed using the PROMIS Early Childhood Parent Report Short Form v1.0 – Anxiety 8a (April 1, 2021). This parent-reported instrument captures observable anxiety-related behaviors and symptoms in young children. It includes 8 items scored on a 5-point response scale and similarly generates standardized T-scores.

PROMIS measures have demonstrated strong psychometric properties, including good internal consistency, construct validity, and responsiveness across pediatric and adult populations. Use of PROMIS instruments allows harmonization with other clinical trials and facilitates cross-study comparisons through standardized T-score metrics.

2. Numeric Rating Scale for Anxiety (NRS-A): In addition to PROMIS measures, situational anxiety will be assessed using a Numeric Rating Scale for Anxiety (NRS-A). Participants (or parents reporting for young children when appropriate) will rate current anxiety on an 11-point scale from:

- 0 = No anxiety at all
- 10 = Worst possible anxiety

The NRS-A provides a rapid assessment of real-time anxiety intensity and is suitable for use in clinical settings where brevity is required. It has been widely used in peri-procedural and acute care settings and correlates with longer validated anxiety instruments.

Administration Plan

- Parents will complete the PROMIS Anxiety 8a at protocol-defined time points.
- Children ≥8 years of age who are developmentally able will complete the self-report PROMIS Anxiety 8a.
- For children 4–7 years of age, parents will complete the PROMIS Early Childhood Parent Report Anxiety 8a.
- The NRS-A will be administered at predefined peri-procedural time points to capture acute state anxiety.
- All measures may be administered electronically (e.g., REDCap) or via paper forms per site preference.

Rationale: The use of PROMIS instruments ensures standardized, psychometrically robust assessment of anxiety across developmental stages while minimizing respondent burden. The addition of the NRS-A allows efficient capture of acute anxiety changes relevant to procedural timing within the TEAPOT study.

## 8.3 Adverse Events and Serious Adverse Events

### 8.3.1 Definition of Adverse Events (AE)

Adverse event means any untoward medical occurrence associated with the use of an intervention in humans, whether or not considered intervention-related (21 CFR 312.32 (a)).

### 8.3.2 Definition of Serious Adverse Events (SAE)

An adverse event (AE) or suspected adverse reaction is considered "serious" if, in the view of either the investigator or sponsor, it results in any of the following outcomes: death, a life-threatening adverse event, inpatient hospitalization, prolongation of existing hospitalization, a persistent or significant incapacity or substantial disruption of the ability to conduct normal life functions, or a congenital anomaly/birth defect. Important medical events that may not result in death, be life-threatening or require hospitalization may be considered serious when, based upon appropriate medical judgment, they may jeopardize the participant and may require medical or surgical intervention to prevent one of the outcomes listed in this definition. Examples of such medical events include allergic bronchospasm requiring intensive treatment in an emergency room or at home, blood dyscrasias or convulsions that do not result in inpatient hospitalization, or the development of drug dependency or drug abuse. All serious, unexpected, and related adverse events that are unresolved at the time of the subject's termination from the study will be followed by the Clinical Center investigators until the events are resolved, the subject is lost to follow-up, the adverse event is otherwise explained, or has stabilized.

### 8.3.3 Classification of an Adverse Event

#### 8.3.3.1 Severity of Event

For adverse events (AEs) not included in the protocol-defined grading system, the following guidelines will be used to describe severity.

**A. Mild** – Events require minimal or no treatment and do not interfere with the participant's daily activities.

**B. Moderate** – Events result in a low level of inconvenience or concern with the therapeutic measures. Moderate events may cause some interference with functioning.

**C. Severe** – Events interrupt a participant's usual daily activity and may require systemic drug therapy or other treatment. Severe events are usually potentially life-threatening or incapacitating. Of note, the term “severe” does not necessarily equate to “serious”.

#### 8.3.3.2 Relationship to Study Intervention

All adverse events (AEs) must have their relationship to study intervention assessed by the clinician who examines and evaluates the participant based on temporal relationship and his/her clinical judgment. The degree of certainty about causality will be graded using the categories below. In a clinical trial, the study product must always be suspect.

**Probably Related** – There is evidence to suggest a definitive or causal relationship, and the influence of other factors is unlikely. The clinical event, including an abnormal laboratory test result, occurs within a reasonable time after administration of the study intervention, is unlikely to be attributed to concurrent disease or other drugs or chemicals, and follows a clinically reasonable response on withdrawal.

**Potentially Related** – There is some evidence to suggest a causal relationship (e.g., the event occurred within a reasonable time after administration of the trial medication). However, other factors may have contributed to the event (e.g., the participant's clinical condition, other concomitant events). Although an AE may rate only as “possibly related” soon after discovery, it can be flagged as requiring more information and later be upgraded to “probably related” or “definitely related”, as appropriate. This can also include a clinical event, including an abnormal laboratory test result, whose temporal relationship to study intervention administration makes a causal relationship improbable (e.g., the event did not occur within a reasonable time after administration of the study intervention) and in which other drugs or chemicals or underlying disease provide plausible explanations (e.g., the participant's clinical condition, other concomitant treatments).

**Not Related** – A clinical event, including an abnormal laboratory test result, whose temporal relationship to study intervention administration makes a causal relationship improbable (e.g., the event did not occur within a reasonable time after administration of the study intervention) and in which other drugs or chemicals or underlying disease provide plausible explanations (e.g., the participant's clinical condition, other concomitant treatments). The AE is completely independent of study intervention administration, and/or evidence exists that the event is definitely related to another etiology. There must be an alternative, definitive etiology documented by the clinician.

#### 8.3.3.3 Expectedness

An AE will be considered unexpected if the nature, severity, or frequency of the event is not consistent with the risk information previously described for the study intervention.

### 8.3.4 Time Period and Frequency for Event Assessment and Follow-Up

The occurrence of an adverse event (AE) or serious adverse event (SAE) may come to the attention of study personnel during study visits and interviews of a study participant presenting for medical care, or upon review by a study monitor. All serious, unexpected, and related adverse events that are unresolved at the time of the subject's termination from the study will be followed by the Clinical Center investigators until the events are resolved, the subject is lost to follow-up, the adverse event is otherwise explained, or has stabilized.

All AEs including local and systemic reactions not meeting the criteria for SAEs will be captured on the appropriate case report form (CRF). Information to be collected includes event description, time of onset, clinician's assessment of severity, relationship to study product (assessed only by those with the training and authority to make a diagnosis), and time of resolution/stabilization of the event. All AEs occurring while on study must be documented appropriately regardless of relationship. All AEs will be followed to an adequate resolution.

Any medical condition that is present at the time that the participant is screened will be considered as a baseline and not reported as an AE. However, if the study participant's condition deteriorates at any time during the study, it will be recorded as an AE.

Changes in the severity of an AE will be documented to allow an assessment of the duration of the event at each level of severity to be performed. AEs characterized as intermittent require documentation of onset and duration of each episode.

Study staff will record all reportable events with start dates occurring any time after informed consent is obtained for 24 hours for non-serious AEs and 7 days for SAEs after randomization and at the 30-day (+/- 5 days) follow-up phone call for any AEs.  At each study visit, the investigator will inquire about the occurrence of AE/SAEs since the last visit.  Events will be followed for outcome information until resolution or stabilization.

### 8.3.5 Adverse Event Reporting

Assuring patient safety is an essential component of this protocol. Each participating site investigator will have primary responsibility for the safety of the individual patients under his or her care. All adverse events occurring after study randomization for 30 days (+/- 5 days) will be recorded and entered the electronic data entry system into UTHSCSA REDCap for single site study and to the REDCap Database provided by the DCC for the multisite study. In accordance with local IRB requirements, the site investigator may be required to report such events to the IRB in addition to notifying the DCC.

### 8.3.6 Serious Adverse Event Reporting

The study clinician will immediately report to the sponsor any serious adverse event, whether considered study intervention-related, including those listed in the protocol or investigator brochure, and must include an assessment of whether there is a reasonable possibility that the study intervention caused the event. Study endpoints that are serious adverse events (e.g., all-cause mortality) must be reported by following the protocol unless there is evidence suggesting a causal relationship between the study intervention and the event (e.g., death from anaphylaxis). In that case, the investigator must immediately report the event to the sponsor.

All serious adverse events (SAEs) will be followed until satisfactory resolution or until the site investigator deems the event to be chronic or the participant is stable. Other supporting documentation of the event may be requested by the Data Coordinating Center (DCC)/study sponsor and should be provided as soon as possible.

The study sponsor will be responsible for notifying the Food and Drug Administration (FDA) of any unexpected fatal or life-threatening suspected adverse reaction as soon as possible, but in no case later than 7 calendar days after the sponsor's initial receipt of the information. In addition, the sponsor must notify FDA and all participating investigators in an Investigational New Drug (IND) safety report of potentially serious risks, from clinical trials or any other source, as soon as possible, but in no case later than 15 calendar days after the sponsor determines that the information qualifies for reporting.

### 8.3.7 Reporting Events to Participants

All serious, unexpected, and related adverse events that are unresolved at the time of the patient's termination from the study or discharge from the hospital will be followed by the Clinical Center investigators until the events are resolved, the patient is lost to follow-up, the adverse event is otherwise explained or has stabilized.

## 8.4 Unanticipated Problems

### 8.4.1 Definition of Unanticipated Problems (UP)

The Office for Human Research Protections (OHRP) considers unanticipated problems involving risks to participants or others to include, in general, any incident, experience, or outcome that meets **all** the following criteria:

A. Unexpected in terms of nature, severity, or frequency is given (a) the research procedures that are described in the protocol-related documents, such as the institutional Review Board (IRB)-approved research protocol and informed consent document; and (b) the characteristics of the participant population being studied.

B. Related or possibly related to participation in the research (“possibly related” means there is a reasonable possibility that the incident, experience, or outcome may have been caused by the procedures involved in the research); and

C. Suggests that the research places participants or others at a greater risk of harm (including physical, psychological, economic, or social harm) than was previously known or recognized.

### 8.4.2 Unanticipated Problem Reporting

The investigator will report unanticipated problems (UPs) to the reviewing Institutional Review Board (IRB) and to the Data Coordinating Center (DCC)/lead principal investigator (PI). The UP report will include the following information:

A. Protocol identifying information: protocol title and number, PI's name, and the IRB project number.

B. A detailed description of the event, incident, experience, or outcome.

C. An explanation of the basis for determining that the event, incident, experience, or outcome represents a UP;

D. A description of any changes to the protocol or other corrective actions that have been taken or are proposed in response to the UP.

To satisfy the requirement for prompt reporting, UPs will be reported using the following timeline:

A. UPs that are serious adverse events (SAEs) will be reported to the IRB and to the DCC/study sponsor within a timeline in accordance with the policy of the investigator becoming aware of the event.

B. Any other UP will be reported to the IRB and to the DCC/study sponsor within the timeline in accordance with the policy of the investigator becoming aware of the problem.

C. All UPs should be reported to appropriate institutional officials (as required by an institution's written reporting procedures), the supporting agency head (or designee), and the Office for Human Research Protections (OHRP) within a timeline in accordance with policy, of the IRB's receipt of the report of the problem from the investigator.

### 8.4.3 Reporting Unanticipated Problems to Participants

All serious, unexpected, and related adverse events that are unresolved at the time of the patient's termination from the study or discharge from the hospital will be followed by the research staff until the events are resolved, the patient is lost to follow-up, the adverse event is otherwise explained or has stabilized. If the local IRB determines an unanticipated problem to pose harm to the participating patients, the site investigator will contact the patient with a letter and a phone call. This letter will describe the unanticipated problem and if any follow-up or additional care is needed.

# 9 Statistical Considerations

## 9.1 Statistical Hypotheses

The pilot study is not designed to test an efficacy hypothesis, but rather to determine the feasibility for patient enrollment and the number of doses received per patient.

**Our primary hypothesis** for the single site and the multi-site study is that we can enroll at least 0.6 patients per site per month and deliver a minimum of two out of three planned nebulized TXA doses to >90% of enrolled patients.  The single site study will only be one year and the multi-site study for 18 months. We will test each of this (yes/no) hypothesis using a Mantel Haenszel test stratified by site for a total of 12 patients for the single-site study for greater than 24 nebulization and for the multi-site study up to 22 patients with greater than 44 nebulization's recorded as delivered.

**Our secondary goals** will be to determine if nebulized TXA prevents going to the OR for PTH and utilization of blood products. These goals are not powered to be tested in the pilot study. **Our second aim** is to verify a pulmonary physiological based pharmacokinetic (PBPK) model (i.e., nasal cavity, pharynx, and lungs) will predict the change in the area under the curve (AUC0-α) of TXA concentration in systemic compartments with accuracy (average absolute fold error <2) and precision (average fold error <2). We will calculate the AUC of accuracy and precision of the model using the linear trapezoid rule. For the PBPK model, we will use previously published PK data to construct the model before verifying it with systematic samples.

## 9.2 Sample Size Determination

Multiple studies suggest that 76% of children with PTH go to the OR for surgical control of their hemorrhage. This pilot study sample size is based on the goals for the subsequent phase 3 trial. Conservatively, we estimate that nebulized TXA will decrease the incidence of surgical care for PTH from 76% to 61%, or in other words a 15% effect size.  The sample size for phase 3 trial based on an effect size of 0.15 with randomization of 324 children with PTH using an a priori, one-tailed, Fishers exact test stratified by site, with 80% power, alpha=0.025. This trial will then need to enroll at least 0.6 patients per site per month at 12 sites over 4 years (0.563 patients x 12 sites x 48 months = 345 patients). Thus, the go/no-go threshold for the subsequent phase 3 trial is an enrollment goal of 0.6 patients per site per month in this pilot study. For the single site trial designed for one year, we hope to enroll 12 patients. For the mutli-site study we hope to enroll 22 patients’ across 3 enrolling sites over 12 months at 0.6 patients x 3 sites x 12 months = 21.6, rounded up to 22 targeted enrollments.

## 9.3 Populations for Analyses

A. Received nebulized TXA

B. Received nebulized saline (placebo control)

## 9.4 Statistical Analyses

### 9.4.1 General Approach

The pilot trial is not powered to evaluate efficacy. Feasibility, efficacy, and safety outcomes will undergo descriptive analysis.

### 9.4.2 Analysis of the Primary Efficacy Endpoint(s)

Differences between TXA and saline conditions with respect to the primary outcome (i.e., need for surgical intervention: yes or no) will be analyzed statistically using the Mantel Haenszel test stratified by site.

### 9.4.3 Analysis of the Secondary Endpoint(s)

Our primary secondary outcome is to select the PBPK model that best characterizes the data using individual and population acceptance criteria. To apply the acceptance criteria, we will simulate plasma drug concentrations for everyone using the PBPK model and will compare predictions with observed concentrations. We will assess model bias by calculating the average fold error between predicted and observed concentrations: AFE=10[1/N∑log(predicted/observed)]. The AFE indicates model under- (AFE<1) or over-prediction (AFE>1) when compared with observed values of the individual. We will assess model precision by calculating the absolute average fold error: AAFE= 10[1/N∑|log(predicted/observed) |] for each curve. We will consider <30% bias (AFE 0.7–1.3) and ≤2-AAFE reasonable predictions. We will assess population predictability by generating a prediction interval (5th to 95th percentile) of drug concentrations per time point for the population and quantifying the number of observed concentrations that fall outside of the prediction interval. The model will be accepted if 90% of observed concentrations fall within the prediction interval.

Differences in exploratory outcomes will be analyzed by unpaired t-tests (for ratio measures such as blood loss) or by Mann-Whiney tests (for ordinal measures such as parental anxiety scale). However, the pilot study is not designed to determine a difference in these outcomes but to determine the feasibility and procedures of the main trial.

### 9.4.4 Safety Analyses

Incidences of seizure or thromboembolic events will be recorded for up to 30 (+/-5) days after receipt of study drug to be collected by e-mail, phone, or text for discharged patients and by chart review for patients who remain in the hospital. Differences in safety outcomes will be analyzed by unpaired t-tests (for ratio measures such as blood loss) or by Mann-Whitney tests.

### 9.4.5 Baseline Descriptive Statistics

Baseline differences in PTH risk factors including age, weight/BMI, insurance, race, and gender will be evaluated between TXA and placebo.

# 10 Supporting Documentation and Operational Considerations

## 10.1 Regulatory, Ethical, and Study Oversight Considerations

### 10.1.1 Informed Consent Process

Consent forms describing in detail the study intervention, study procedures, and risks are given to the participant and written, or electronic documentation of informed consent is required prior to starting intervention/administering study intervention.  The following consent materials will be submitted to the UTHSCSA for the single site study and Utah Single IRB for the multi-site study: English and Spanish written and electronic consents.

#### 10.1.1.1 Consent/assent and Other Informational Documents Provided to participants

All patients scheduled for tonsillectomy at study sites will receive study information in the clinic, pre-surgical packets, and by e-mail upon discharge. Information on the consent and assent process for children will be provided.

#### 10.1.1.2 Consent Procedures and Documentation

**Informed Consent:** We will enroll eligible patients who are between 2 and 17 years of age.  Advertisements and notifications of the study will be provided to parents as best as possible during the pre-operative or operative visits for a tonsillectomy to inform them about the study.

We will attempt to obtain written informed consent at the time of patient eligibility if the parent or guardian is available and able to provide informed consent. After determining that a subject is eligible, and the parent or guardian is present in the ED and able to provide informed consent, the site investigator or designee will approach the parent or guardian to go over participation for their child in the study (Figure 2). The parent or guardian will be informed about the goals of the study, the study procedures, and the potential risks and benefits of participation. If the parent or guardian refuses permission for their child to take part, then all clinical management will be provided by the clinical staff in accordance with institutional practice and judgment.

Under FDA Regulation 21 CFR 50.55(e)(1), where clinical investigations are more than minimal risk but presenting the prospect of direct benefit to individual subjects (CFR 50.52) and permission is to be obtained from guardians, the permission of one guardian is sufficient. Participants must be informed that participation is voluntary and that they may withdraw from the study at any time, without prejudice. A copy of the informed consent document will be given to the participants for their records.  The informed consent process will be conducted and documented in the source document (including the date), and the form signed before the participant undergoes any study-specific procedures. The rights and welfare of the participants will be protected by emphasizing to them that the quality of their medical care will not be adversely affected if they decline to participate in this study.

Under FDA Regulation 21 CFR 50.55(c)(2), because the intervention holds out a prospect of direct benefit that is important to the health or well-being of the child and is available only in the context of the clinical investigation, assent of the child is not a necessary condition for enrollment in the study.

**Subject Consent:** Subjects who are eligible for this study must be younger than 18 years of age at the time of enrollment. If a subject reaches the age of 18 years after enrollment but during the study period, then informed consent of the patient becomes applicable. If this occurs, 18-year-old subjects who are alert and competent, and capable of giving consent will be asked, following an appropriate discussion of risks and benefits, to give consent to the study for further study procedures. We will obtain informed consent from these subjects for continuation in the study. Continued participation will consist of a telephone follow-up call, considered to be minimal risk. Subject consent will be waived if the subject has a severely reduced mental age, decreased level of consciousness, psychological problems, or other legitimate reasons as judged by the Institutional Review Board.

**Subject Assent:** Assent should be obtained from a child who is able to understand trial and what they are required to do, which the investigators designate as greater than 7 years of age. The child should be given an explanation, at a level appropriate to the child's age, maturity, and condition, of the procedures to be used, their meaning to the child in terms of discomfort and inconvenience, and the general purpose of the research. In addition to providing the informed consent document to the parent or legal guardian, an information sheet should be developed for the appropriate age range of the child for his or her easier comprehension. Informed consent should be documented on the consent form, using the Surrogate Signature Section. The parent, guardian, or representative signs and personally dates the consent form as the Person Giving Consent, and the assent of the child, when appropriate, is documented by having the child sign as the Subject.

### 10.1.2 Study Discontinuation and Closure

This study may be temporarily suspended or prematurely terminated if there is sufficient reasonable cause.  Written notification, documenting the reason for study suspension or termination, will be provided by the suspending, or terminating party to study participants, investigator, funding agency, the Investigational New Drug (IND) sponsor and regulatory authorities.  If the study is prematurely terminated or suspended, the Sponsor/Principal Investigator (PI) will promptly inform study participants, the IRB, and the FDA and will provide the reason(s) for the termination or suspension.  Study participants will be contacted, as applicable, and be informed of changes to the study visit schedule.

Circumstances that may warrant termination or suspension include, but are not limited to:

- Determination of unexpected, significant, or unacceptable risk to participants
- Demonstration of efficacy that would warrant stopping.
- Insufficient compliance to protocol requirements
- Data that are not sufficiently complete and/or valuable
- A determination that the primary endpoint has been met
- Determination of futility

The study may resume once concerns about safety, protocol compliance, and data quality are addressed, and satisfy the sponsor, IRB, and/or Food and Drug Administration (FDA).

### 10.1.3 Confidentiality and Privacy

All research activities will be conducted in as private a setting as possible. Participant confidentiality and privacy is strictly held in trust by the participating investigators, their staff, and the sponsor(s) and their interventions. This confidentiality is extended to cover the testing of biological samples and genetic tests in addition to the clinical information relating to participants. Therefore, the study protocol, documentation, data, and all other information generated will be held in strict confidence. No information concerning the study or the data will be released to any unauthorized third party without the prior written approval of the sponsor.

The study monitor, other authorized representatives of the sponsor, representatives of the Institutional Review Board (IRB), regulatory agencies, or study product may inspect all documents and records required to be maintained by the investigator, including but not limited to, medical records (office, clinic, or hospital) and pharmacy records for the participants in this study. The clinical study site will permit access to such records.

The study participant's contact information will be securely stored for internal use during the study. At the end of the study, all records will continue to be kept in a secure location for as long a period as dictated by the reviewing IRB, Institutional policies, or requirements.

Study participant research data, which is for purposes of statistical analysis and scientific reporting, for the single site study will be stored on the UTHSCSA REDCap (please see Data Quality Assurance plan) and for the multi-site study will be transmitted to and stored at the University of Utah Data Coordinating Center. This will not include the participant's contact or identifying information. Rather, a unique study identification number will identify individual participants and their research data. The study data entry and study management systems used by clinical sites and by University of Utah Data Coordinating Center research staff will be secured and password protected. At the end of the study, all study databases will be de-identified and archived at the UTHSCA Redcap or the University of Utah Data Coordinating Center.

### 10.1.4 Future Use of Stored Specimens and Data

Data collected for the single-site study will be analyzed and stored at the UTHSCSA REDCap. Data collected for the multi-site study will be analyzed and stored at the University of Utah Data Coordinating Center. After the study is completed, the de-identified, archived data (left over pharmacokinetic samples and other de-identified study data) will be turned over to BioLINCC, for use by other researchers including those outside of the study. This data and BioLINCC (Biologic Specimen and Data Repository Information Coordinating Center) is a clinical specimen and study database funded and supported by the National Heart Lung and Blood Institute ([www.biolincc.nhlbi.nih.gov](http://www.biolincc.nhlbi.nih.gov)). When the study is completed, access to study data and/or samples will be provided through the BIOLINCC.

### 10.1.5 Key Roles and Study Governance

| **Principal Investigator** |
| --- |
| Andrew D. Meyer, MD, MS |
| U.T. Health Science Center |
| 7703 Floyd Curl Drive |
| San Antonio, TX 78229 |

### 10.1.6 Safety Oversight

**Data Safety Monitor: For the single site clinical trial, the PI and study team will select an individual at the institution who** is typically an expert in clinical research, biostatistics, or a relevant medical field who is not otherwise involved in the trial’s design or execution. The monitor will receive periodic reports of adverse events, participant enrollment and retention data, and any emerging trends suggesting increased risk or benefit. They evaluate whether additional safety measures or protocol modifications are needed, ensuring the trial adheres to regulatory requirements and upholds the highest ethical standards for the protection of participants.

The rationale for a data safety monitor for the single site trial is the scale and complexity of the study are typically smaller, making it feasible for one qualified individual to thoroughly review safety and data integrity without undue burden or bias. This single data safety monitor can maintain direct, real-time oversight of all study activities and participant outcomes since everything takes place in one location. However, in a multi-site trial, the complexity expands exponentially: data are generated from different locations, involving larger and often more diverse participant populations, varying clinical practices, and multiple teams. This increased scope necessitates a broader range of expertise, as well as a mechanism to ensure uniform standards of safety monitoring and unbiased assessment across all sites. Consequently, a formal Data Safety Monitoring Board (DSMB)—composed of multiple experts from relevant fields—is required to collectively review, interpret, and act upon safety and efficacy data from each site, providing more comprehensive oversight and ensuring that potential risks are identified and managed consistently.

**Data Safety Monitoring Board (DSMB)**: A DSMB will be selected and approved by the funding agency for the study. The DSMB will be composed of a minimum of 5 members. The membership will include representation from experts in the fields of pediatric otolaryngology, biostatistics, bioethics, emergency medicine, and pediatric critical care. The DSMB will have a charter, will approve the protocol prior to implementation, and will review interim analyses as applicable.

The purpose of the DSMB is to advise the sponsors and principal investigator(s) regarding the continuing safety of study subjects and the continuing validity and scientific merit of the study. The DSMB is responsible for monitoring the accrual of study subjects, adherence to the study protocol, assessments of data quality, the performance of individual Clinical Centers, review of serious adverse events, and other subject safety issues.

For the multi-site trial, the DCC will send reports relating to these topics to DSMB members prior to each DSMB meeting. The DCC will staff the DSMB meetings and produce minutes of open sessions. Minutes of closed or executive sessions of the DSMB will be produced and retained by the DSMB Chairperson. These closed minutes will not be available outside the DSMB prior to the end of the study. The DSMB Chairperson will prepare a summary of each DSMB meeting that conveys the public conclusions of the DSMB, with respect to protocol alterations and recommendations concerning the continuation of the study. When applicable, this will be sent directly to the study sponsor for approval before it is provided to the DCC and sites. When the summary is provided to the DCC, the DCC will send the summary to all Clinical Center investigators for submission to their respective Institutional Review Boards/Research Ethics Board(s).

**Frequency of Interim Analysis: The Data Safety Monitor will meet with the study team at the the start of the study and every 6 participants enroll**ed for a total of 3 visits. The DSMB will be expected to meet every six months, including an initial meeting prior to the start of subject enrollment as well as after enrollment of the 10th subject (anticipated 2-3 months after the onset of subject enrollment). The DSMB, however, will have the discretion to alter meeting timing and frequency.

**Conflict of Interest:** As described in the DSMB charter, Data Safety Monitor and DSMB members should be independent of all entities sponsoring, organizing, conducting, or regulating the TEAPOT study. Specifically, Data Safety Monitor and DSMB members should not have any significant financial interest in the study's conduct or outcome, nor be involved in the design of this study. Additionally, members must disclose any actual or potential conflicts of interest involving pharmaceutical or biotechnology companies and contract research organizations. This includes any financial arrangement, consultancy agreement (direct or via the third party), research support, or any other relationship that could be construed as introducing potential bias to their role as a DSMB member.

The Data Safety Monitor, DSMB, and sponsor official will be responsible for determining whether any consultancies or financial interests of a member may be viewed as potentially materially impacting their objectivity. This decision is to be based on the reasonable belief that objectivity is in doubt. Each DSMB member and the Data Safety monitor is responsible for informing the UTHSCA or University Utah Compliance Office, FDA and DSMB Chairperson if any relevant changes in financial interest or other developments affecting potential or perceived conflict of interest developed during the duration of DSMB membership.

### 10.1.7 Clinical Monitoring

Clinical site monitoring is conducted to ensure that the rights and well-being of trial participants are protected, that the reported trial data are accurate, complete, and verifiable, and that the conduct of the trial is in compliance with the currently approved protocol/amendment(s), with International Conference on Harmonization Good Clinical Practice (ICH GCP), and with applicable regulatory requirement(s). For the single site study, the UTHSCSA Research Quality Administration (RAQ) will conduct site visits to ensure compliance. For the multi-site study the Data Coordinating Center in conjunction with the sponsor Dr. Meyer at UTHSCSA will conduct site visits to ensure compliance.

**Electronic Data Capture System**

For the single site study, please see the Data Quality Assurance Plan where we described the use of UTHSCSA Redcap for the electronic data capture system. For the multi-site study, the Data Coordinating Center will create the electronic data capture (EDC) system and worksheets that can be used by clinical site research coordinators and investigators. Data will be entered via the Web into the EDC. Worksheets and study documents will be maintained in locked filing cabinets in locked offices at each site. The Data Coordinating Center currently uses OpenClinica, REDCap, and XNAT as its data capture systems; this may be changed at any time without requiring a protocol amendment.

**Study Monitoring**

The investigators recognize the importance of ensuring data of excellent quality. Site monitoring is critical to this process. Site monitoring has been a very effective tool for maintaining data quality in previous PECARN studies, and we will utilize this process to ensure excellent quality data in the proposed study. Our site monitoring plan is designed to identify problems with sites and methods for handling problems that arise. Site monitors must be provided with full access to study materials and the medical records for study subjects. If the medical records are in electronic form, the clinical investigator or an authorized individual must provide any assistance necessary to facilitate the site monitor's review of data in the electronic medical record.

**Site Monitoring Plan**

For the single site study, the UTHSCSA RAQ office perform an initial site visit, then provides monitoring after four participants are enrolled, at the completion of 12 enrollments, and additional visits as necessary. For the multi-site study, a supplemental study-special monitoring plan separate from the protocol will be completed which outlines special criteria for monitoring. This plan will include the number of planned site visits, criteria for focused visits, or additional visits, a plan for chart review, and a follow-up plan for non-compliant sites. The monitoring plan also describes the type of monitoring that will take place (e.g., a sample of all subjects within a site; key data, or all data), the schedule of visits, how they are reported, and a time frame to resolve any issues found. Remote site monitoring activities will be determined by the Data Coordinating Center and UTHSCSA RAQ in coordination with the study principal investigators and based on Risk Assessment and Risk Management processes in accordance with ICH GCP guidelines.

**Clinical Site Monitoring**

Site monitoring visits will be performed by a trained site monitor during the study period to ensure regulatory compliance, patient safety, and to monitor the quality of data collected. Essential document binders, regulatory documents, and data collection forms may be reviewed. Interim visits will take place depending on grant budget, site enrollment, and compliance issues identified. The site monitor will provide each site with a written report, and sites will be required to follow up on any deficiencies. It is anticipated that the study monitoring visits for this protocol will consist of a site initiation visit (prior to patient enrollment), interim visits, and a close-out visit. The site initiation may take place as group training made up of site investigators and research assistants.

**Remote Monitoring**

The UTHSCSA RAQ and the Data Coordinating Center may supplement on-site monitoring with remote monitoring activities. Remote monitoring involves a detailed review of the data entered by the Clinical Center and consultations with the Clinical Center investigator and/or research coordinator to review safety and data quality. This may require uploading de-identified copies of special parts of the medical record, subject study file, regulatory documentation, or other source documents to the UTHSCSA RAQ and the Data Coordinating Center staff, who review those materials against the data recorded in the electronic data capture system. This helps assure protocol compliance and accurate data collection. The Data Coordinating Center may conduct more remote monitoring activities early in the trial to assure protocol compliance and identify any training issues that may exist. Remote monitoring of the documents will be retained in accordance with federal requirements. The safety of subjects will be monitored and ensured in accordance with the Data and Safety Monitoring Board (DSMB) plan.

**Pharmacy Monitoring**

The site investigational pharmacy must maintain adequate records of all dispensed study drug. Each site pharmacy will be monitored and may be requested to send copies of these documents to the UTHSCA RAQ and the Data Coordinating Center.

### 10.1.8 Quality Assurance and Quality Control

**Study Training:** A formal training program for investigators and research staff will be held prior to the start of enrollment. The training program will cover regulatory topics and Good Clinical Practice. The training will also provide in-depth explanations regarding study procedures, clinical care, adverse event reporting, data entry procedures, quality assurance, site monitoring, and the informed consent process. A manual of operations will be provided to each investigator prior to the start of enrollment. The manual will detail specific information about the study procedures, regulatory information, safety reporting, and other necessary information. Updates and revisions to the manual will be made available electronically. For the single site study, the study team and Dr. Meyer will be main contact for questions. The Data Coordinating Center, in collaboration with the principal investigators (Drs. Meyer and Nishijima), will be the main contact for study questions.

A meeting will be held to review study activities, study workflow, and data entry procedures. Each site investigator should instruct the group of ED physicians at their home institutions about the study and serve as local advocates and champions for the study and answer questions as they arise. Throughout the study, the study team will also have conference calls, webinars, and in-person meetings to update on study progress and provide ongoing training.

**PECARN Network Involvement**: If selected for funding the study will be use PECARN site for the multi-site study. This proposed pilot study of nebulized TXA for children with post-tonsillectomy hemorrhage will be completed within PECARN. PECARN is the first and only federally funded pediatric emergency medicine research network in the United States, initially funded in 2001 through cooperative agreements between academic medical centers and the Health Resources Services Administration / Maternal and Child Health Bureau / Emergency Medical Services for Children Program (HRSA / MCHB / EMSC). PECARN was created to address barriers to research in emergency medical services for children (EMSC), including the lack of infrastructure. PECARN conducts high-priority, multi-institutional research on the prevention and management of acute illnesses and injuries in children. Currently, PECARN is made up of 7 research nodes containing three Hospital Emergency Department Affiliates (HEDAs) and one EMS Affiliate. The EMS Research Node has three EMS Affiliates. PECARN sites consist of academic, community, urban, general, and children's hospitals. The PECARN network Emergency Departments serve approximately 1.3 million acutely ill and injured children every year and the 9 EMS affiliates account for more than 113,000 pediatric runs annually. For more information, please see <https://pecarn.org/about/> .

PECARN is governed by a Steering Committee that formulates and monitors policies and procedures guiding all research activities and reviews and approves research proposals. All major scientific decisions are made by a majority vote. Subcommittees, including the Protocol Review and Development Subcommittee (PRADS), the Grant Writing and Publications Subcommittee (GAPS), the Feasibility and Budget Subcommittee (FAB), the Quality, Safety, and Regulatory Affairs Subcommittee (QUASI), and the Research Coordinator Advisory Committee (RCAC) carry out specific tasks identified by the Steering Committee. This pilot project has been reviewed and approved by PECARN.

**Quality Assurance (QA)**: Each clinical site will perform internal quality management of study conduct, data, and biological specimen collection, documentation, and completion.  An individualized quality management plan will be developed to describe a site's quality management.

**Quality control (QC)** procedures will be implemented beginning with the data entry system and data QC checks that will be run on the database will be generated. Any missing data or data anomalies will be communicated to the site(s) for clarification/resolution. Following written Standard Operating Procedures (SOPs), the monitors will verify that the clinical trial is conducted, and data are generated, and biological specimens are collected, documented (recorded), and reported in compliance with the protocol, International Conference on Harmonisation Good Clinical Practice (ICH GCP), and applicable regulatory requirements (e.g., Good Laboratory Practices (GLP), Good Manufacturing Practices (GMP)).

The investigational site will provide direct access to all trial-related sites, source data/documents, and reports for the purpose of monitoring and auditing by the sponsor, and inspection by local and regulatory authorities.

### 10.1.9 Data Handling and Record Keeping

#### 10.1.9.1 Data Collection and Management Responsibilities

**Single-Site Data Collection**

**For the single-site trial,** data collection will be the responsibility of the clinical trial staff into the UTHSCSA REDCap system at the site under the supervision of the site investigator (please see data quality assurance plan). All data will be recorded in the electronic case report form (eCRF) as source documents. When not able to record documents into REDCap system, hardcopies of the study visit worksheets will be provided for use as source document worksheets for recording data for each participant consented/enrolled in the study concistent with eCRF. Clinical data (including adverse events (AEs), concomitant medications, and expected adverse reactions data) and clinical laboratory data will be entered into UTHSCSA RedCap, data capture system provided by the UT Health. Since UTHSCSA RedCap is not 21 CFR Part 11 compliant, please see data quality assurance plan and request to the FDA for waiver to use UTHSCA REDCap. Data for the single center trial will not be used for new indication submission to the FDA. The data system includes password protection and internal quality checks, such as automatic range checks, to identify data that appear inconsistent, incomplete, or inaccurate. Clinical data will be entered directly from the source documents.

**Data Center Description**

The Data Coordinating Center (DCC) in the Department of Pediatrics at the University of Utah School of Medicine provides data coordination and management services for a variety of national research networks. Anchoring these services is a new state-of-the-art, energy-efficient data center completed in 2013. The data center facility supports more than 1400 users around the world and provides a secure, reliable, enterprise-wide infrastructure for delivering critical DCC systems and services. The new data center was built using high industry standards and energy-efficient cooling solutions. The data center is cooled by Rittal's LCP inline cooling technology, providing efficiency, redundancy, and modularity. Cooling is based upon a hot/cold aisle design that allows for even air distribution with minimal hot spots. The data center electrical power system contains a redundant Mitsubishi uninterruptible power system (UPS) with a diesel backup generator. The data center is protected with an FM200 fire suppression system, early warning smoke detectors, and a heat detection warning system to act as a secondary system to the smoke detectors. Security guards are on-site conducting access control and rounds 24/7/365. Entry into the data center is restricted by card access and layered security measures and controls. The data center and external building access points are monitored with video surveillance.

In 2011 the data center began a large-scale VMware server virtualization deployment. Currently, the data center has virtualized about 99% of its environment. The virtual environment consists of more than 200 virtual servers. The data center's virtualization solution provides key advantages:

- high availability in the event of hardware failure, virtual servers automatically go back online in a seamless process.
- flexible infrastructure: disk storage, memory, and processor capacity can be increased or reallocated at any time.
- rapid deployment: servers can be provisioned on demand with minimal waiting on the hardware of software.

The data center also enhanced its storage resources by implementing a networked storage system to support its virtualized environment. The data center currently manages over 50 terabytes of data. The storage solution consists of Dell's EqualLogic PS Series Storage system for providing a virtualized storage area network (SAN). Some of the benefits that are realized through this technology are:

- storage architecture is no longer be a bottleneck for IT services.
- performance is better than with the previous architecture.
- tiered storage is now possible.
- provisioning and reclamation of SAN disk will be much easier; and most importantly, the new architecture includes a redesign of the SAN fabric to include complete redundancy.

Production servers running critical applications are clustered and configured for failover events. Servers are backed up with encryption through a dedicated backup server that connects across an internal 10-gigabit network to a tape drive. DCC storage area networking (SAN) applications, clusters, and switch-to-switch links are also on a 10-gigabit network. Incremental backups occur hourly Monday through Friday from 6 am to 6 pm. Incremental backups also are performed each night with full system backups occurring every Friday. Tapes are stored in a fireproof safe inside the data center facility, and full backups are taken off-site on a weekly basis to an off-site commercial storage facility.

In the event of catastrophic failures, such as a fire in the server facility, daily backups would probably survive because of the fire suppression system and fireproof safe, but there would be an obvious delay in re-establishing data center function because the servers will not survive such a disaster. Destruction of the data center facility could cause the loss of up to one week's data. In future investments, the data center is making co-location, disaster recovery, and business continuity solutions a top priority.

DCC information systems are available 24 hours a day, 7 days a week to all users unless a scheduled maintenance interruption is required. If this occurs, we notify all users of the relevant systems, and data entry can be deferred until after the interruption is over. Critical systems availability has exceeded 99.9% for the past two years, and there has been no unscheduled downtime in over five years.

**Security and Confidentiality**

The data center coordinates the network infrastructure and security with the Health Sciences Campus (HSC) information systems at the University of Utah. This provides us with effective firewall hardware, automatic network intrusion detection, and the expertise of dedicated security experts working at the University. Network equipment includes four high-speed switches. User authentication is centralized with two Windows 2012 domain servers. Communication over public networks is encrypted with virtual point-to-point sessions using transport layer security (TLS) or virtual private network (VPN) technologies, both of which provide at least 128-bit encryption. All of our Web-based systems use the TLS protocol to transmit data securely over the Internet. Direct access to data center machines is only available while physically located inside our offices, or via a VPN client.

All network traffic is monitored for intrusion attempts, security scans are regularly run against our servers, and our IT staff is notified of intrusion alerts. Security is maintained with Windows 2012 user/group domain-level security. Users are required to change their passwords every 90 days, and workstations time out after 5 minutes of inactivity. All files are protected at group and user levels; database security is handled in a similar manner with group-level access to databases, tables, and views in Microsoft SQL Server. Finally, all laptop computers in use in the DCC or in the Department of Pediatrics are whole disk encrypted.

The data center uses control center tools to continuously monitor systems and failure alerts. Environmental and network systems are also monitored to ensure uptime. Highly trained system administrators on staff are available to respond to high-risk emergency events.

All personnel involved with the DCC have signed confidentiality agreements concerning data encountered in the course of their daily work. All personnel (including administrative staff) have received Human Subjects Protection and Health Information Portability and Accountability Act (HIPAA) education. We require all users to sign special agreements concerning security, confidentiality, and the use of our information systems before access is provided.

**Record Access**

The medical record and study files (including informed consent, permission, and assent documents) must be made available to authorized representatives of the UTHSCSA study team and Data Coordinating Center, upon request, for source verification of study documentation. In addition, medical information and data generated by this study must be available for inspection upon request by representatives (when applicable) of the Food and Drug Administration (FDA), NIH, other Federal funders, and the Institutional Review Board (IRB) for each study site.

#### 10.1.9.2 Study Records Retention

Study documents should be retained for a minimum of 2 years after the last approval of a marketing application in an International Conference on Harmonization (ICH) region and until there are no pending or contemplated marketing applications in an ICH region or until at least 2 years have elapsed since the formal discontinuation of clinical development of the study intervention. These documents should be retained for a longer period, however, if required by local regulations. No records will be destroyed without the written consent of the sponsor, if applicable. It is the responsibility of the sponsor to inform the investigator when these documents no longer need to be retained.

### 10.1.10 Protocol Deviations

A protocol deviation is any noncompliance with the clinical trial protocol, International Conference on Harmonisation Good Clinical Practice (ICH GCP), or MOP requirements. The noncompliance may be either on the part of the participant, the investigator, or the study site staff. As a result of deviations, corrective actions are to be developed by the site and implemented promptly.

These practices are consistent with ICH GCP:

- 4.5 Compliance with Protocol, sections 4.5.1, 4.5.2, and 4.5.3
- 5.1 Quality Assurance and Quality Control, section 5.1.1
- 5.20 Noncompliance, sections 5.20.1, and 5.20.2.

It is the responsibility of the site investigator to use continuous vigilance to identify and report deviations within 5 working days of identification of the protocol deviation, or within 10 working days of the scheduled protocol-required activity.  All deviations must be addressed in study source documents, reported to the Grant Sponsor and the University of Utah Data Coordinating Center.  Protocol deviations must be sent to the reviewing IRB per their policies. The site investigator is responsible for knowing and adhering to the reviewing IRB requirements. Further details about the handling of protocol deviations will be included in the MOP.

### 10.1.11 Publication and Data Sharing Policy

This study will be conducted in accordance with the following publication and data sharing policies and regulations:

National Institutes of Health (NIH) Public Access Policy, which ensures that the public has access to the published results of NIH-funded research. It requires scientists to submit final peer-reviewed journal manuscripts that arise from NIH funds to the digital archive PubMed Central upon acceptance for publication.

This study will comply with the NIH Data Sharing Policy and Policy on the Dissemination of NIH-Funded Clinical Trial Information and the Clinical Trials Registration and Results in Information Submission rule. As such, this trial will be registered at ClinicalTrials.gov, and results in information from this trial will be submitted to ClinicalTrials.gov. In addition, every attempt will be made to publish results in peer-reviewed journals.  Data from this study may be requested from other researchers 3 years after the completion of the primary endpoint by contacting Andrew D. Meyer, MD, MS or through the DCC public use dataset website. More information can be obtained at https://medicine.utah.edu/pediatrics/research/dcc/

### 10.1.12 Conflict of Interest Policy

The independence of this study from any actual or perceived influence, such as by the pharmaceutical industry, is critical. Therefore, any actual conflict of interest of persons who have a role in the design, conduct, analysis, publication, or any aspect of this trial will be disclosed and managed. Furthermore, persons who have a perceived conflict of interest will be required to have such conflicts managed in a way that is appropriate to their participation in the design and conduct of this trial.  The study leadership in conjunction with the National Heart Lung and Blood Institute has established policies and procedures for all study group members to disclose all conflicts of interest and will establish a mechanism for the management of all reported dualities of interest.

## 10.2 Additional Considerations

### 10.2.1 FOOD AND DRUG ADMINISTRATION

This trial is being conducted under an Investigational New Drug application allowed to proceed by the Food and Drug Administration (Investigational New Drug application #145749). The clinical investigator and any study team members who will assist the investigator and make a direct and significant contribution to the date at each participating site will complete a Form FDA 1572, “Statement of Investigator."

### 10.2.2 HEALTH INSURANCE PORTABILITY AND ACCOUNTABILITY ACT

Data elements collected include the date of birth and date of admission. Before statistical analyses, dates will be used to calculate subject age at the time of the study events. The final data sets (used for study analyses and archived at the end of the study) will be de-identified and will exclude these specific dates. Data elements for race, ethnicity, and gender are also being collected. These demographic data are required for Federal reporting purposes to delineate subject accrual by race, ethnicity, and gender.

For purposes of the DCC handling potential protected health information (PHI) and producing the de-identified research data sets that will be used for analyses, all study sites have been offered a Business Associate Agreement with the University of Utah. Copies of executed Business Associate Agreements are maintained at the DCC.

### 10.2.3 INCLUSION OF WOMEN AND MINORITIES

There will be no exclusion of subjects based on gender, race, or ethnicity.

### 10.2.4 CLINICALTRIALS.GOV REQUIREMENTS

This trial will be registered at ClinicalTrials.gov in accordance with Federal regulations.

## 10.3 Abbreviations

| AE | Adverse Event |
| --- | --- |
| ANCOVA | Analysis of Covariance |
| CFR | Code of Federal Regulations |
| CLIA | Clinical Laboratory Improvement Amendments |
| CMP | Clinical Monitoring Plan |
| COC | Certificate of Confidentiality |
| CONSORT | Consolidated Standards of Reporting Trials |
| CRF | Case Report Form |
| DCC | Data Coordinating Center |
| DHHS | Department of Health and Human Services |
| DSMB | Data Safety Monitoring Board |
| DRE | Disease-Related Event |
| EC | Ethics Committee |
| ED | Emergency Department |
| eCRF | Electronic Case Report Forms |
| FDA | Food and Drug Administration |
| FDAAA | Food and Drug Administration Amendments Act of 2007 |
| FFR | Federal Financial Report |
| GCP | Good Clinical Practice |
| GLP | Good Laboratory Practices |
| GMP | Good Manufacturing Practices |
| GWAS | Genome-Wide Association Studies |
| HIPAA | Health Insurance Portability and Accountability Act |
| IB | Investigator’s Brochure |
| ICH | International Conference on Harmonization |
| ICMJE | International Committee of Medical Journal Editors |
| IDE | Investigational Device Exemption |
| IND | Investigational New Drug Application |
| IRB | Institutional Review Board |
| ISM | Independent Safety Monitor |
| ISO | International Organization for Standardization |
| ITT | Intention-To-Treat |
| LSMEANS | Least-squares Means |
| MedDRA | Medical Dictionary for Regulatory Activities |
| MOP | Manual of Procedures |
| MSDS | Material Safety Data Sheet |
| NCT | National Clinical Trial |
| NIH | National Institutes of Health |
| NIH IC | NIH Institute or Center |
| OHRP | Office for Human Research Protections |
| PI | Principal Investigator |
| PTH | Post-tonsillectomy hemorrhage |
| QA | Quality Assurance |
| QC | Quality Control |
| SAE | Serious Adverse Event |
| SAP | Statistical Analysis Plan |
| SMC | Safety Monitoring Committee |
| SOA | Schedule of Activities |
| SOC | System Organ Class |
| SOP | Standard Operating Procedure |
| TXA | Tranexamic Acid |
| UP | Unanticipated Problem |
| US  UTHSCSA | United States  University of Texas Health Science Center at San Antonio |

## 10.4 Protocol Amendment History

| **Version** | **Date** | **Description of Change** | **Brief Rationale** |
| --- | --- | --- | --- |
| 0.25 | April 2021 | Updates after meetings with DCC | Improvement to aims and protocol |
| 0.30 | June 2021 | Updates from Nodes | Improvement to protocol |
| 0.4 | Oct 2021 | Updates from first protocol | Improvement to protocol |
| 0.5 | Dec 2021 | Updates from 2nd protocol | Improvement to protocol |
| 1.0 | Jan 2024 | Preparation for grant and FDA submission | Improvement to protocol |
| 1.1 | Mar 2024 | Added 30-day AE follow-up | FDA recommendations |
| 2.0 | Jan 2025 | Updated the scheduling of specimen collections section, revised the safety oversight section, data quality system. | IRB approval, University Health System approval, and Data quality assurance section. |
| 3.0 | Feb 27 2026 | Updated date and version, funding source, change of anxiety scale from STAI to PROMIS, updated background paragraph, condensed the study visits from 0, 1, 7, and 30 to 0-1, 7 and 30, eliminated pain and anxiety from day 0-1. | To improve feasibility and reduce participant burden while maintaining the scientific objectives of the study. Updated part of background section with recent literature, clarifying funding sources, updating surveys, reduce the number and complexity of research assessments during the ED visit to shorten survey completion time and minimizing burden on participants during an acute medical event. |
|  |  |  |  |
|  |  |  |  |
|  |  |  |  |
|  |  |  |  |
|  |  |  |  |
|  |  |  |  |
|  |  |  |  |
|  |  |  |  |
|  |  |  |  |

# 11 References

### References

1. Subramanyam R, Varughese A, Willging JP, Sadhasivam S, Future of pediatric tonsillectomy and perioperative outcomes., Int J Pediatr Otorhinolaryngol. 2013 Feb;77(2):194-9

[Pmid:23159321](https://www.ncbi.nlm.nih.gov/PubMed/23159321)

1. Bhattacharyya N, Lin HW, Changes and consistencies in the epidemiology of pediatric adenotonsillar surgery, 1996-2006., Otolaryngol Head Neck Surg. 2010 Nov;143(5):680-4

[Pmid:20974339](https://www.ncbi.nlm.nih.gov/PubMed/20974339)

1. Patel HH, Straight CE, Lehman EB, Tanner M, Carr MM, Indications for tonsillectomy: a 10 year retrospective review., Int J Pediatr Otorhinolaryngol. 2014 Dec;78(12):2151-5

[Pmid:25447951](https://www.ncbi.nlm.nih.gov/PubMed/25447951)

1. Clark CM, Schubart JR, Carr MM, Trends in the management of secondary post-tonsillectomy hemorrhage in children., Int J Pediatr Otorhinolaryngol. 2018 May;108:196-201

[Pmid:29605354](https://www.ncbi.nlm.nih.gov/PubMed/29605354)

1. Whelan RL, Shaffer A, Anderson ME, Hsu J, Jabbour N, Reducing rates of operative intervention for pediatric post-tonsillectomy hemorrhage., Laryngoscope. 2018 Aug;128(8):1958-1962

[Pmid:29314032](https://www.ncbi.nlm.nih.gov/PubMed/29314032)

1. Liu L, Rodman C, Worobetz NE, Johnson J, Elmaraghy C, Chiang T, Topical biomaterials to prevent post-tonsillectomy hemorrhage., J Otolaryngol Head Neck Surg. 2019 Sep 6;48(1):45

[Pmid:31492172](https://www.ncbi.nlm.nih.gov/PubMed/31492172)

1. Crabtree G, Dobie RA, The effect of unilateral corneal anesthesia on the Schirmer test., Otolaryngol Head Neck Surg. 1989 Jun;100(6):631-2

[Pmid:2501743](https://www.ncbi.nlm.nih.gov/PubMed/2501743)

1. Lee SY, Chong S, Balasubramanian D, Na YG, Kim TK, What is the Ideal Route of Administration of Tranexamic Acid in TKA? A Randomized Controlled Trial., Clin Orthop Relat Res. 2017 Aug;475(8):1987-1996

[Pmid:28283902](https://www.ncbi.nlm.nih.gov/PubMed/28283902)

1. Bafaqih H, Chehab M, Almohaimeed S, Thabet F, Alhejaily A, AlShahrani M, Zolaly MA, Abdelmoneim AA, Abd ES, Pilot trial of a novel two-step therapy protocol using nebulized tranexamic acid and recombinant factor VIIa in children with intractable diffuse alveolar hemorrhage., Ann Saudi Med. 2015 May-Jun;35(3):231-9

[Pmid:26409798](https://www.ncbi.nlm.nih.gov/PubMed/26409798)

1. Schwarz W, Ruttan T, Bundick K, Nebulized Tranexamic Acid Use for Pediatric Secondary Post-Tonsillectomy Hemorrhage., Ann Emerg Med. 2019 Mar;73(3):269-271

[Pmid:30292524](https://www.ncbi.nlm.nih.gov/PubMed/30292524)

1. Burk CD, Miller L, Handler SD, Cohen AR, Preoperative history and coagulation screening in children undergoing tonsillectomy., Pediatrics. 1992 Apr;89(4 Pt 2):691-5

[Pmid:1557263](https://www.ncbi.nlm.nih.gov/PubMed/1557263)

1. Arora R, Saraiya S, Niu X, Thomas RL, Kannikeswaran N, Post tonsillectomy hemorrhage: who needs intervention?, Int J Pediatr Otorhinolaryngol. 2015 Feb;79(2):165-9

[Pmid:25547960](https://www.ncbi.nlm.nih.gov/PubMed/25547960)

1. Erwin DZ, Heichel PD, Wright LM BS, Goldstein NA, McEvoy TP, Earley MA, Meyer AD, Post-tonsillectomy hemorrhage control with nebulized tranexamic acid: A retrospective cohort study., Int J Pediatr Otorhinolaryngol. 2021 Aug;147:110802

[Pmid:34146910](https://www.ncbi.nlm.nih.gov/PubMed/34146910)

1. Maeda T, Michihata N, Sasabuchi Y, Matsui H, Ohnishi Y, Miyata S, Yasunaga H, Safety of Tranexamic Acid During Pediatric Trauma: A Nationwide Database Study., Pediatr Crit Care Med. 2018 Dec;19(12):e637-e642

[Pmid:30199511](https://www.ncbi.nlm.nih.gov/PubMed/30199511)

1. El Rassi E, de Alarcon A, Lam D, Practice patterns in the management of post-tonsillectomy hemorrhage: An American Society of Pediatric Otolaryngology survey., Int J Pediatr Otorhinolaryngol. 2017 Nov;102:108-113

[Pmid:29106855](https://www.ncbi.nlm.nih.gov/PubMed/29106855)

1. Ker K, Edwards P, Perel P, Shakur H, Roberts I, Effect of tranexamic acid on surgical bleeding: systematic review and cumulative meta-analysis., BMJ. 2012 May 17;344:e3054

[Pmid:22611164](https://www.ncbi.nlm.nih.gov/PubMed/22611164)

1. Koizumi M, Ishimaru M, Matsui H, Fushimi K, Yamasoba T, Yasunaga H, Tranexamic acid and post-tonsillectomy hemorrhage: propensity score and instrumental variable analyses., Eur Arch Otorhinolaryngol. 2019 Jan;276(1):249-254

[Pmid:30402793](https://www.ncbi.nlm.nih.gov/PubMed/30402793)

1. Baugh RF, Archer SM, Mitchell RB, Rosenfeld RM, Amin R, Burns JJ, Darrow DH, Giordano T, Litman RS, Li KK, Mannix ME, Schwartz RH, Setzen G, Wald ER, Wall E, Sandberg G, Patel MM, American Academy of Otolaryngology-Head and Neck Surgery Foundation., Clinical practice guideline: tonsillectomy in children., Otolaryngol Head Neck Surg. 2011 Jan;144(1 Suppl):S1-30

[Pmid:22996082](https://www.ncbi.nlm.nih.gov/PubMed/22996082)

1. Chan CC, Chan YY, Tanweer F, Systematic review and meta-analysis of the use of tranexamic acid in tonsillectomy., Eur Arch Otorhinolaryngol. 2013 Feb;270(2):735-48

[Pmid:30321510](https://www.ncbi.nlm.nih.gov/PubMed/30321510)

1. Wand O, Guber E, Guber A, Epstein Shochet G, Israeli-Shani L, Shitrit D, Inhaled Tranexamic Acid for Hemoptysis Treatment: A Randomized Controlled Trial., Chest. 2018 Dec;154(6):1379-1384

[Pmid:30375746](https://www.ncbi.nlm.nih.gov/PubMed/30375746)

1. González-Quevedo T, Larco JI, Marcos C, Guilarte M, Baeza ML, Cimbollek S, López-Serrano MC, Piñero-Saavedra M, Rubio M, Caballero T, Management of Pregnancy and Delivery in Patients With Hereditary Angioedema Due to C1 Inhibitor Deficiency., J Investig Allergol Clin Immunol. 2016;26(3):161-7

Pmid:23448586

1. Xie J, Lenke LG, Li T, Si Y, Zhao Z, Wang Y, Zhang Y, Xiao J, Preliminary investigation of high-dose tranexamic acid for controlling intraoperative blood loss in patients undergoing spine correction surgery., Spine J. 2015 Apr 1;15(4):647-54

[PMID:25457470](https://www.ncbi.nlm.nih.gov/PubMed/25457470)

1. Guo J, Gao X, Ma Y, Lv H, Hu W, Zhang S, Ji H, Wang G, Shi J, Different dose regimes and administration methods of tranexamic acid in cardiac surgery: a meta-analysis of randomized trials., BMC Anesthesiol. 2019 Jul 15;19(1):129

[PMID:31307381](https://www.ncbi.nlm.nih.gov/PubMed/31307381)

1. Zilinsky I, Barazani TB, Visentin D, Ahuja K, Martinowitz U, Haik J, Subcutaneous Injection of Tranexamic Acid to Reduce Bleeding During Dermatologic Surgery: A Double-Blind, Placebo-Controlled, Randomized Clinical Trial., Dermatol Surg. 2019 Jun;45(6):759-767

[PMID:30640775](https://www.ncbi.nlm.nih.gov/PubMed/30640775)

1. Ausen K, Pleym H, Liu J, Hegstad S, Nordgård HB, Pavlovic I, Spigset O, Serum Concentrations and Pharmacokinetics of Tranexamic Acid after Two Means of Topical Administration in Massive Weight Loss Skin-Reducing Surgery., Plast Reconstr Surg. 2019 Jun;143(6):1169e-1178e

[PMID:31136475](https://www.ncbi.nlm.nih.gov/PubMed/31136475)
