# Supplementary material for: TranExamic Atomized for Pediatric post-Operative Tonsillectomy hemorrhage (TEAPOT): Study protocol for a pilot randomized controlled trial
Source: PLoS One. 2026 Jul 28;21(7):e0353841. doi: 10.1371/journal.pone.0353841 (PMC13411930; doi:10.1371/journal.pone.0353841)
Supplement: S3 File — (DOCX) [file pone.0353841.s003.docx]

**WHO Trial Registration Data Set**

1. **Primary Registry and Trial Identifying Number:** ClinicalTrials.gov ID# NCT07565753
2. Date of Registration in Primary Registry: May 4^th^, 2026
3. Secondary Identifying Numbers: none
4. Source(s) of Monetary or Material Support: NHLBI 1R34HL177446-01A1 and UT Health Clinical Pilot Award
5. Primary Sponsor: University of Texas Health at San Antonio IND holder
6. Secondary Sponsor: University of Utah Data Coordinating Center Central IRB holder
7. Contact for Public Queries: Stephanie Perez, MHA, (210) 450-8973, [perezs11@uthscsa.edu](mailto:perezs11@uthscsa.edu?subject=NCT06580509,%20STUDY00000638,%20TranExamic%20Atomized%20for%20Pediatric%20Post-Operative%20Tonsillectomy%20Hemorrhage)
8. Contact for Scientific Queries: Andrew D Meyer, MD, MS, 210-567-4424, [meyera@uthscsa.edu](mailto:meyera@uthscsa.edu)
9. Public Title: TEAPOT Pilot Trial
10. Scientific Title: TranExamic Atomized for Pediatric post-Operative Tonsillectomy hemorrhage
11. Countries of Recruitment: United States (University Hospital/University of Texas Health Science Center at San Antonio, San Antonio, Texas; UC Davis Children’s Hospital, Sacramento, California; Hasbro Children’s Hospital, Providence, Rhode Island).
12. Health Condition(s) or Problem(s) Studied: Post-Tonsillectomy Hemorrhage
13. Intervention(s): The study intervention involves randomized to either nebulized tranexamic acid (TXA) or saline to pediatric patients with secondary post tonsillectomy hemorrhage (PTH). The intervention consists of three consecutive doses of 500 mg/5 mL nebulized TXA or 5 ml of saline.
14. Key Inclusion and Exclusion Criteria: Inclusion criteria include children between age of 2 to 17 years of age that receive a tonsillectomy and presents to the ED with secondary post-tonsillectomy hemorrhage. Exclusion criteria include known and documented bleeding or clotting disorder, known pregnancy, the patient is a ward of the state, patients with known hypersensitivity or allergic response to tranexamic acid, parents or guardians that cannot communicate in English or Spanish, intubation prior to enrollment, and previously enrolled patients.
15. Study Type: A multi-center, randomized, double-blinded, placebo-controlled trial. It is a parallel arms pilot trial randomizing up to 22 patients.
16. Anticipated Date of First enrollment: July 1^st^, 2026
17. Sample Size: 22 patients
18. Recruitment status: Pending
19. Primary Outcome: 1. Evaluate the feasibility to enroll 0.6 patients/site/month, 2. Nebulize at least two of three doses of TXA to children with PTH, and 3. Determine the local and systemic concentrations of nebulized TXA in children.
20. Secondary Outcomes: 1. Determine the number and rationale for PTH patients to return to the operating room (OR), 2. Determine the number of blood transfusions and estimated blood loss, 3. Determine the recurrence of PTH after TXA, saline, or return to the OR, and 4. Establish pain and anxiety after receiving study intervention.
21. Ethics Review: Approved by the University of Utah Institutional Review Board (IRB_00191036) on October 29^th^, 2025, with protocol amended on April 9^th^, 2026
22. Completion Date, Summary Results: pending
23. IPD Sharing Statement: As this a feasibility study there is no plan to share individual clinical trial participant-level data using University of Utah Data Coordinating Center.
